# Phase separation of SPIN1 through its IDR facilitates histone methylation readout and tumorigenesis

**DOI:** 10.1093/jmcb/mjae024

**Published:** 2024-05-22

**Authors:** Yukun Wang, Yuhan Chen, Mengyao Li, Jiayue Wang, Yuhan Jiang, Rong Xie, Yifeng Zhang, Zhihua Li, Zhenzhen Yan, Chen Wu

**Affiliations:** College of Life Sciences, Hebei University, Baoding 071002, China; College of Life Sciences, Hebei University, Baoding 071002, China; College of Life Sciences, Hebei University, Baoding 071002, China; College of Life Sciences, Hebei University, Baoding 071002, China; College of Life Sciences, Hebei University, Baoding 071002, China; College of Life Sciences, Hebei University, Baoding 071002, China; College of Life Sciences, Hebei University, Baoding 071002, China; College of Life Sciences, Hebei University, Baoding 071002, China; College of Life Sciences, Hebei University, Baoding 071002, China; College of Life Sciences, Hebei University, Baoding 071002, China

**Keywords:** SPIN1, IDR, phase separation, histone methylation reader, tumorigenesis

## Abstract

Spindlin1 (SPIN1) is a unique multivalent histone modification reader that plays a role in ribosomal RNA transcription, chromosome segregation, and tumorigenesis. However, the function of the extended N-terminal region of SPIN1 remains unclear. Here, we demonstrated that SPIN1 can form phase-separated and liquid-like condensates both *in vitro* and *in vivo* through its N-terminal intrinsically disordered region (IDR). The phase separation of SPIN1 recruits the histone methyltransferase MLL1 to the same condensates and enriches the H3K4 methylation marks. This process also facilitates the binding of SPIN1 to H3K4me3 and activates tumorigenesis-related genes. Moreover, SPIN1-IDR enhances the genome-wide chromatin binding of SPIN1 and facilitates its localization to genes associated with the MAPK signaling pathway. These findings provide new insights into the biological function of the IDR in regulating SPIN1 activity and reveal a previously unrecognized role of SPIN1-IDR in histone methylation readout. Our study uncovers the crucial role of appropriate biophysical properties of SPIN1 in facilitating gene expression and links phase separation to tumorigenesis, which provides a new perspective for understanding the function of SPIN1.

## Introduction

Phase separation has emerged as a fundamental mechanism that regulates various biological processes, and biomolecular condensates driven by phase separation can compartmentalize and concentrate biochemical reactions within cells (Banani et al., 2017; Alberti et al., 2019). Accumulating evidence indicates that liquid–liquid phase separation (LLPS) is triggered by multivalent interactions mediated by intrinsically disordered regions (IDRs) in proteins (Li et al., 2012; Shin and Brangwynne, 2017). Furthermore, aberrant phase separation and liquid-to-solid phase transition are involved in neurodegenerative diseases, developmental disorders, and cancer development (Alberti and Dormann, 2019; Zhu et al., 2020).

Histone modification patterns and their combinatorial readout have been recognized as a fundamental mechanism for epigenetic regulation. Among the most extensively studied histone post-translational modifications, histone methylation functions as a signal platform for recruiting specific effector molecules containing specialized ‘reader’ domains, such as chromodomain, plant homeodomain, Tudor domain, malignant brain tumor domain, and Pro–Trp–Trp–Pro domain (Yun et al., 2011; Su and Denu, 2016).

Spindlin1 (SPIN1), a highly conserved histone methylation reader in vertebrates (Staub et al., 2002), was initially identified as a meiotic spindle-binding protein in mice (Oh et al., 1997). During interphase, SPIN1 is enriched in the nucleoli of mouse embryonic fibroblasts (Wang et al., 2011). Notably, SPIN1 has been implicated in the progression of human cancer due to its high expression in various cancers, including prostate cancer, breast cancer, pancreatic cancer, colon cancer, and ovarian cancer (Chen et al., 2016, 2018; Fang et al., 2018; Zhou et al., 2021). Moreover, elevated levels of SPIN1 are associated with poor prognosis in patients with breast cancer, colorectal cancer, and gastric cancer (Franz et al., 2015; Fang et al., 2018). Suppressing SPIN1 expression or inhibiting its activity with small molecules leads to the inhibition of tumor cell growth *in vitro* and in xenograft models (Franz et al., 2015; Fang et al., 2018). SPIN1 exerts its oncogenic function by activating multiple cancer-related signaling pathways, such as the PI3K/AKT, Wnt, uL18–MDM2–p53, and RET signaling pathways (Franz et al., 2015; Chen et al., 2016; Fang et al., 2018; Devi et al., 2019). These findings suggest that SPIN1 could serve as a potential target for future anticancer therapies. Furthermore, SPIN1 not only functions as a reader protein for histone methylation, which is involved in regulating downstream gene transcription (Wang et al., 2011, 2012), but also plays a role in chromosome segregation during mitotic or meiotic cell division (Zhang et al., 2008).

Structural studies revealed that SPIN1 consists of three tandem Tudor-like domains; the first Tudor-like domain recognizes H3R8me2a (Su et al., 2014) and the second Tudor-like domain recognizes H3K4me3 (Yang et al., 2012) or H4K20me3 (Shanle et al., 2017; Wang et al., 2018). Recently, Du et al. (2021) provided new structural and functional insights into a previously unappreciated mechanism by which SPIN1 in complex with C11orf84 recognizes H3K4me3 and H3K9me3 dual marks on the same histone H3 tail. In contrast to the well-characterized Tudor-like domains of SPIN1, the function of the unappreciated N-terminal disordered region of SPIN1 remains elusive. Previous studies showed that IDRs enable a broader spectrum of interactions, enhance association kinetics, facilitate catalysis, and promote degradation (Shoemaker et al., 2000; Bemporad et al., 2008; Huang and Liu, 2009). In this study, we investigated the regulatory roles of SPIN1-IDR in H3K4me3 recognition, target gene transcription, and tumorigenesis. We found that human SPIN1 forms phase-separated and liquid-like condensates in the nucleoplasm. The entire N-terminal IDR of SPIN1 is crucial for the formation of these condensates and plays a particularly significant role in regulating SPIN1 LLPS. Furthermore, the regulatory role of SPIN1 in target gene transcription, which involves genome-wide histone recognition, is dependent on SPIN1-IDR. Altogether, our findings reveal that SPIN1 belongs to the growing list of proteins that are regulated by the IDR.

## Results

### SPIN1 forms liquid-like and phase-separated condensates in nuclei

**Figure 1:**
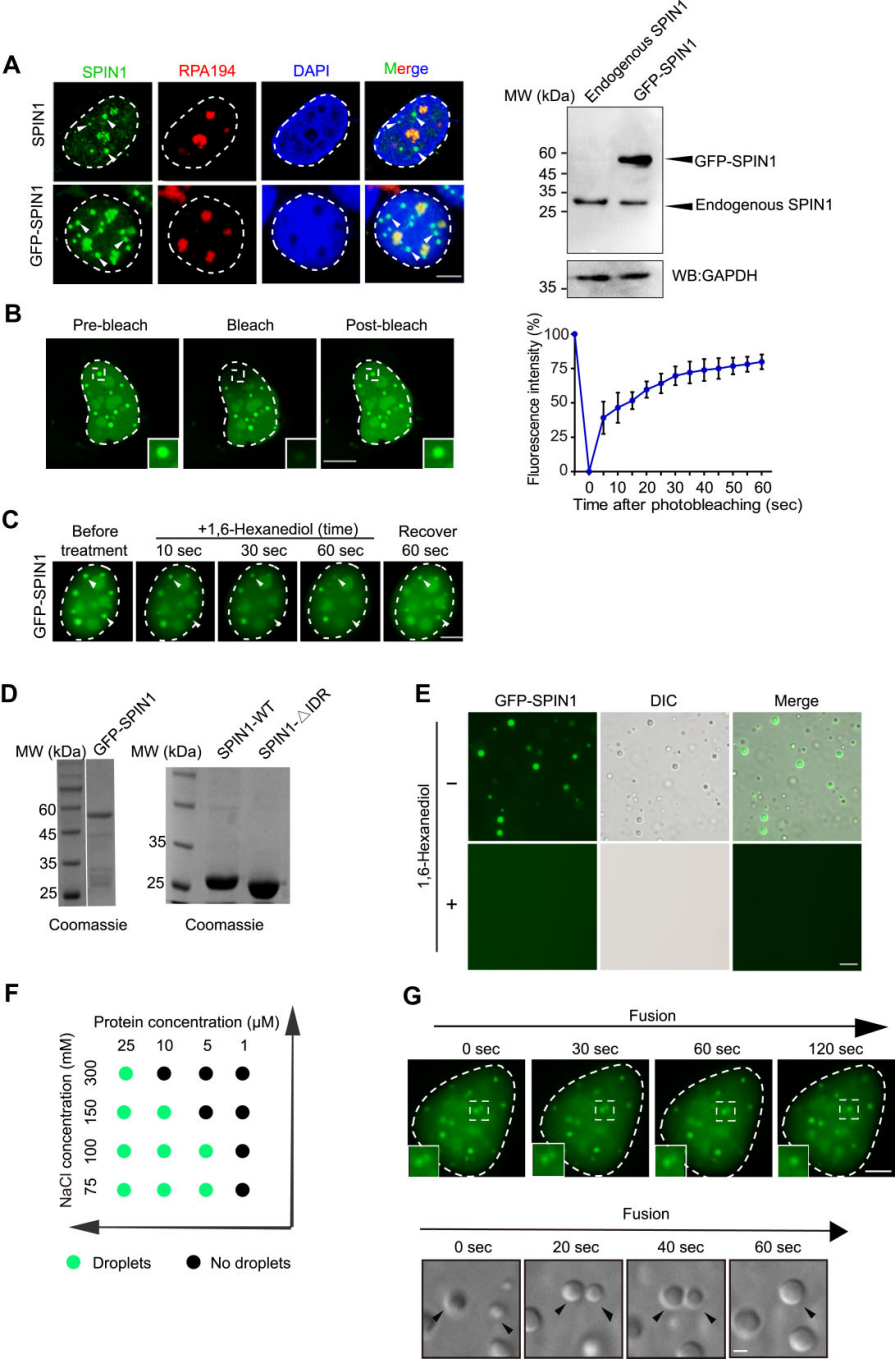
SPIN1 forms reversible, dynamic phase-separated condensates in the nucleus. (**A**) SPIN1 forms condensates in the nucleus. Left: representative co-immunostaining images of endogenous SPIN1 (top) or exogenous GFP-tagged SPIN1 (bottom) with the nucleolus marker RPA194 in U2OS cells. Scale bar, 5 μm. Right: the expression levels of endogenous or exogenous SPIN1. (**B**) Fluorescence recovery kinetics of GFP-SPIN1 analyzed by the FRAP assay. Left: representative images of the condensates formed by SPIN1 before and after photobleaching. The photobleached region is boxed and amplified. Scale bar, 5 μm. Right: statistical analysis of the FRAP assay from three independent experiments. Data are shown as mean ± SEM. (**C**) 1,6-Hexanediol treatment disrupts GFP-SPIN1 droplets. (**D**) GFP-SPIN1, SPIN1-WT, and SPIN1-∆IDR proteins purified from *E. coli* and examined by Coomassie blue staining. (**E**) Images of the droplets formed by 10 μM GFP-SPIN1 in the presence or absence of 2.5% 1,6-hexanediol. Scale bar, 20 μm. (**F**) Phase diagrams of the SPIN1 protein at the concentration ranging from 1 μM to 25 μM in ‘condensation buffer’. (**G**) Representative droplet fusion events at the indicated time points. Rapid fusion of GFP-SPIN1 in U2OS cell nucleus is boxed and amplified (top). The arrowhead indicates the two-droplet fusion *in vitro* (bottom). Scale bar, 5 μm.

SPIN1, which is a unique multivalent epigenetic reader that not only facilitates ribosomal RNA transcription but also regulates additional target gene expression, was reported to be enriched in nucleoli (Wang et al., 2011, 2012). We verified the subcellular localization of SPIN1 in U2OS cells by co-staining endogenous SPIN1 or exogenous GFP-tagged SPIN1 (GFP-SPIN1) with a nucleolar marker RPA194 (Figure 1A). Unexpectedly, in addition to its nucleolar localization, endogenous SPIN1 formed punctate condensates in the nucleoplasm (Figure 1A, indicated by white arrowheads). Exogenous GFP-SPIN1 also localized to nucleoli and gathered as large, rounded condensates in the nucleoplasm (Figure 1A). We observed similar localization pattern of endogenous SPIN1 and exogenous GFP-SPIN1 in SGC7901 cells (Supplementary Figure S1A). Moreover, when we knocked down SPIN1 using small-interfering RNA in U2OS cells, the condensates disappeared (Supplementary Figure S1B). Next, we sought to investigate whether SPIN1 punctate condensates are formed via LLPS using different approaches (Chong et al., 2018; Dao et al., 2018; Sabari et al., 2018; Zhang et al., 2021). First, we performed a fluorescence recovery after photobleaching (FRAP) assay and found that the GFP intensity of SPIN1 condensates rapidly recovered to 60%–70% within 30 sec after photobleaching (Figure 1B), illustrating that the SPIN1 condensates are dynamic. Second, we analyzed the response of the condensates to 2.5% 1,6-hexanediol treatment, which is commonly used to disrupt phase-separated condensates (Chong et al., 2018; Ahn et al., 2021; Duster et al., 2021). Our results showed that 1,6-hexanediol effectively impaired GFP-SPIN1 condensates within 10 sec and disrupted the majority of the condensates within 60 sec, while more than half of the condensates recovered in 60 sec after the removal of 1,6-hexanediol (Figure 1C), indicating that the SPIN1 puncta were highly sensitive to 1,6-hexanediol treatment. Third, by using the purified full-length SPIN1 protein (with an N-terminal GFP tag), we observed that, under *in vitro* conditions, with polyethylene glycol (PEG) as a crowding agent, 10 μM purified SPIN1 spontaneously formed droplets, indicating that purified SPIN1 is capable of undergoing phase separation and displaying liquid-like features. These droplets formed by SPIN1 could be abolished by 1,6-hexanediol treatment (Figure 1D and E). In addition, with increasing SPIN1 concentration, more droplets were formed. We also observed that SPIN1 formed liquid droplets at a low salt concentration (75 mM), and with increasing NaCl concentration, less droplets were formed (Figure 1F; Supplementary Figure S1C). Moreover, the droplets appeared to be able to fuse quite rapidly *in vitro* (within 1 min); consistently, the condensates were able to fuse *in vivo* within 2 min (Figure 1G), suggesting the liquid-like properties and a quick movement of SPIN1 in the condensates. Taken together, these cellular observations indicate that SPIN1 forms liquid-like condensates in the nucleus via LLPS.

### The IDR of SPIN1 is essential for its phase separation

**Figure 2:**
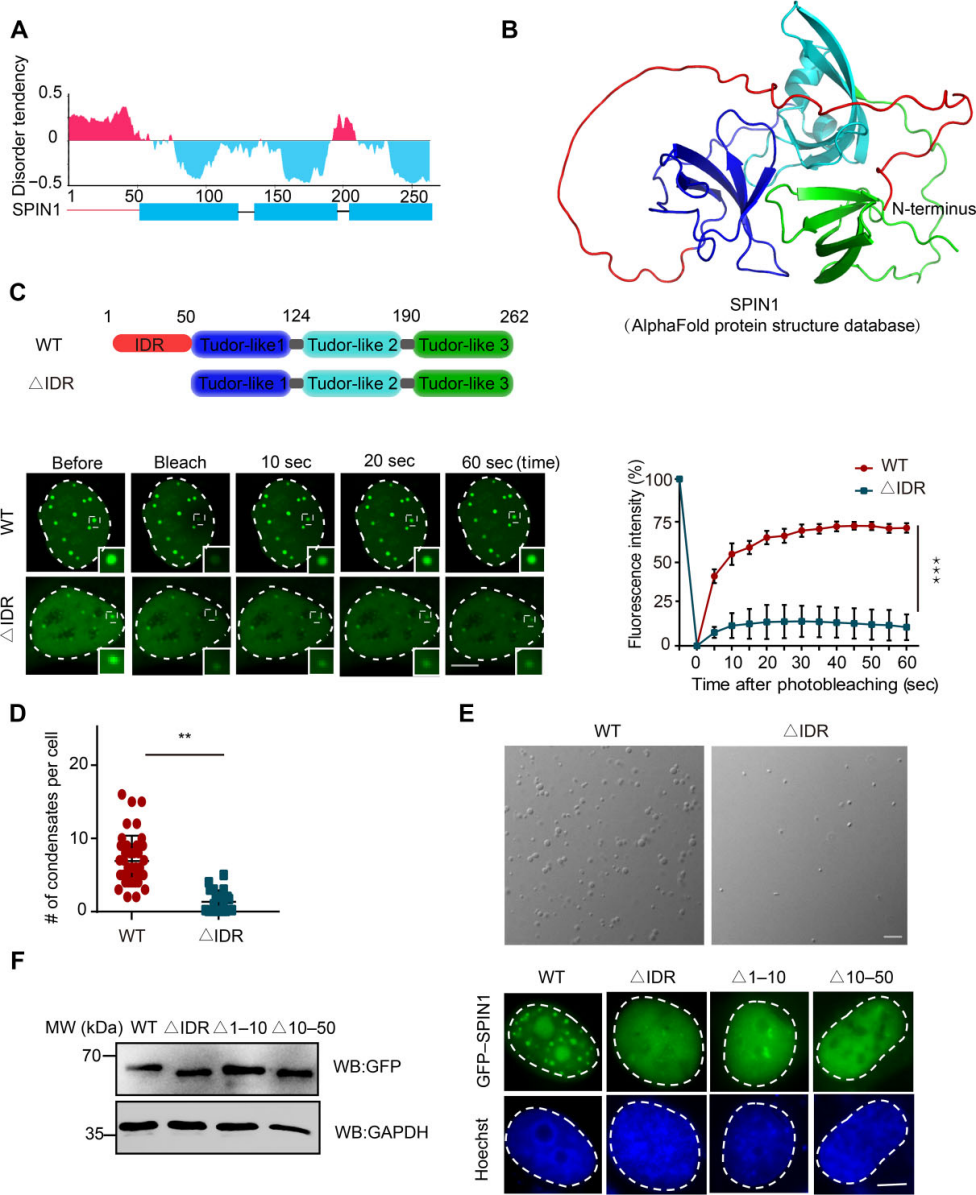
The IDR of SPIN1 is essential for its phase separation. (**A**) The disorder tendency of the SPIN1 protein sequence analyzed by IUPred3. (**B**) The SPIN1 structure analyzed by AlphaFold. SPIN1-IDR is highlighted in red. (**C**) The FRAP assay of condensates formed by GFP-SPIN1-WT or GFP-SPIN1-∆IDR. The schematic diagram on the top shows the domains of SPIN1-WT and the SPIN1-∆IDR mutant. Left: representative images of condensates at the indicated time points. Scale bar, 5 μm. Right: statistical analysis of the FRAP assay from three independent observations. (**D**) Quantification of the condensates formed by GFP-SPIN1-WT or GFP-SPIN1-∆IDR (50 individual cells, mean  ±  SD). (**E**) DIC images of the phase-separated condensates formed by 10 μM SPIN1-WT or SPIN1-∆IDR in the presence of 10% (*w*/*v*) PEG5000. Scale bar, 20 μm. (**F**) Left: the expression levels of SPIN1-WT and SPIN1 mutants. Right: the localization of SPIN1-WT and SPIN1 mutants. Scale bar, 5 μm.

Proteins that undergo LLPS to form punctate condensates often contain IDRs. Accumulating evidence has indicated that the IDR plays a critical role in determining LLPS (Li et al., 2020b, c; Hallegger et al., 2021; Shi et al., 2021). We next sought to identify the domains of SPIN1 that contribute to its condensate formation. The crystal structure of three Tudor-like domains of human SPIN1 at 2.2 Å of resolution (PDB ID: 2NS2) was determined in 2007, revealing three similar well-ordered β-barrel-like structures (Zhao et al., 2007). We analyzed the protein sequence of SPIN1 using IUPred3, an online tool for exploring the evolutionary conservation of disordered proteins coupled with sequence conservation (Erdos et al., 2021). Like most well-documented proteins capable of undergoing LLPS, the N-terminal 50 amino acids of SPIN1 (hereafter referred to as SPIN1-IDR) are highly disordered, while the three Tudor-like domains are well ordered (Figure 2A). We found that SPIN1-IDR is highly flexible, as indicated by the predicted structure of full-length SPIN1 from the AlphaFold Protein Structure Database (Figure 2B). We speculated that this domain might act as a relatively flexible adaptor to assist in the recognition of methylated histone H3, mediated by Tudor-like 1 and Tudor-like 2 domains. Alternatively, the IDR might flexibly bind to other proteins or biomolecules, such as DNA or RNA, to drive condensate formation. To characterize whether the IDR of SPIN1 is responsible for its LLPS, we generated a SPIN1-IDR deletion mutant (SPIN1-∆IDR) and confirmed its expression by western blotting (Supplementary Figure S2C). The FRAP assay revealed that the fluorescence intensity of the condensates formed by GFP-tagged wild-type SPIN1 (SPIN1-WT) rapidly recovered to its original level, whereas the fluorescence intensity of the condensates formed by GFP-tagged SPIN1-∆IDR did not show the same level of recovery (Figure 2C). Moreover, the number and intensity of the condensates formed by SPIN1-∆IDR *in vivo* were remarkably lower compared to SPIN1-WT, indicating reduced SPIN1 dynamicity and impaired puncta formation capacity (Figure 2C and D). Consistently, IDR deletion also affected droplet formation of SPIN1 *in vitro* (Figures 1D and 2E). Furthermore, we replaced SPIN1-IDR with unrelated IDRs from TDP-43 and FUS, which are known to form liquid-like condensates (Hofweber et al., 2018; Hallegger et al., 2021). Compared with SPIN1-∆IDR, both of these chimeras restored the nuclear focus formation and chromatin-binding ability of SPIN1 (Supplementary Figure S2A and B). Then, we investigated the direct involvement of the IDR in chromatin binding. Our results revealed that IDR alone failed to bind to chromatin (Supplementary Figure S2B), consistent with a previous report indicating that SPIN1 is primarily recruited to chromatin through its interaction with H3K4me3 (Zhao et al., 2020). Overall, these results emphasize the critical importance of SPIN1-IDR in facilitating the proper formation of condensates.

To further identify the exact region responsible for the LLPS of SPIN1, we constructed a series of deletion mutants of SPIN1-IDR with a GFP tag on the N-terminus and confirmed their expression by western blotting (Figure 2F; Supplementary Figure S2C). Live-cell imaging experiments revealed that all the deletion mutants abolished the nucleolar localization of SPIN1 and prevented condensate formation (Figure 2F; Supplementary Figure S2C). To exclude the possibility that the N-terminal GFP tag affects the expression and localization of SPIN1, we also generated a series of deletion mutants of SPIN1 with a GFP tag on the C-terminus and obtained similar results (Supplementary Figure S2D and E). These findings highlight the crucial role of the entire IDR in proper LLPS. A previous study showed that the KKR-KK sequence on the N-terminus (amino acids 28–44) of SPIN1 mediated its nucleolar localization and the substitution of KKR-KK with 5A in full-length SPIN1 (SPIN1-5A mutant) completely abolished its nucleolar distribution (Zhang et al., 2018). We also found that the SPIN1-5A mutant failed to localize to the nucleolus but still formed a few nuclear condensates (Supplementary Figure S2D), suggesting that nucleolar localization of SPIN1 is not required for condensate formation.

In addition, SPIN1 is a member of a family of highly related proteins (SPIN1, SPIN2A, SPIN2B, SPIN3, and SPIN4) that all harbor three Tudor-like domains and IDRs (Staub et al., 2002; Bae et al., 2017a; Supplementary Figure S2F). Thus, we asked whether the other members of the SPIN family could form condensates as well. To test this, we ectopically expressed GFP-tagged SPIN family members and quantified the number of condensates formed in one cell and the percentage of cells with condensates. We indeed observed that, similar to SPIN1, both SPIN2A and SPIN3 formed condensates to variable extents, while SPIN4 failed to form condensates (Supplementary Figure S2G and H). Previous studies have indicated that IDRs are typically enriched in particular polar and charged amino acids, including glycine, serine, glutamine, proline, glutamic acid, lysine, and arginine (Romero et al., 2001; Vucetic et al., 2003). We analyzed the amino acid composition of the IDRs in SPIN family members and found that SPIN1-IDR consists of 50 amino acids, including 16 non-polar amino acids and 34 polar amino acids. Notably, polar amino acids account for 68% of the IDR sequence, which is consistent with the characteristic features of IDR sequences. In SPIN2 and SPIN3, polar amino acids make up 72% and 67% of their IDR sequences, respectively. In contrast, SPIN4 has a shorter IDR with only 37 amino acids, of which polar amino acids make up 59% of the IDR sequence (Supplementary Figure S2I). Interestingly, when aligning the IDR sequences of the SPIN family, we observed that SPIN2A, SPIN3, and SPIN4 all lack the amino acids 39–43 compared to SPIN1. Notably, SPIN4 is the only protein that lacks the first nine amino acids (Supplementary Figure S2J). These findings highlight distinct differences in the composition and sequence variations of IDRs among SPIN proteins.

Furthermore, we exchanged the IDRs between SPIN1 and SPIN4 to generate plasmids named SPIN4_IDR_-SPIN1 and SPIN1_IDR_-SPIN4, respectively. Interestingly, the SPIN1_IDR_-SPIN4 chimera formed condensates within the cells, whereas the SPIN4_IDR_-SPIN1 chimera abolished the phase separation property of SPIN1 (Supplementary Figure S2K). Altogether, these findings suggest that the intact IDR of SPIN1 likely plays a key role in mediating SPIN1 LLPS.

### SPIN1-IDR enhances the binding of SPIN1 to H3K4me3 and enriches histone modification

**Figure 3:**
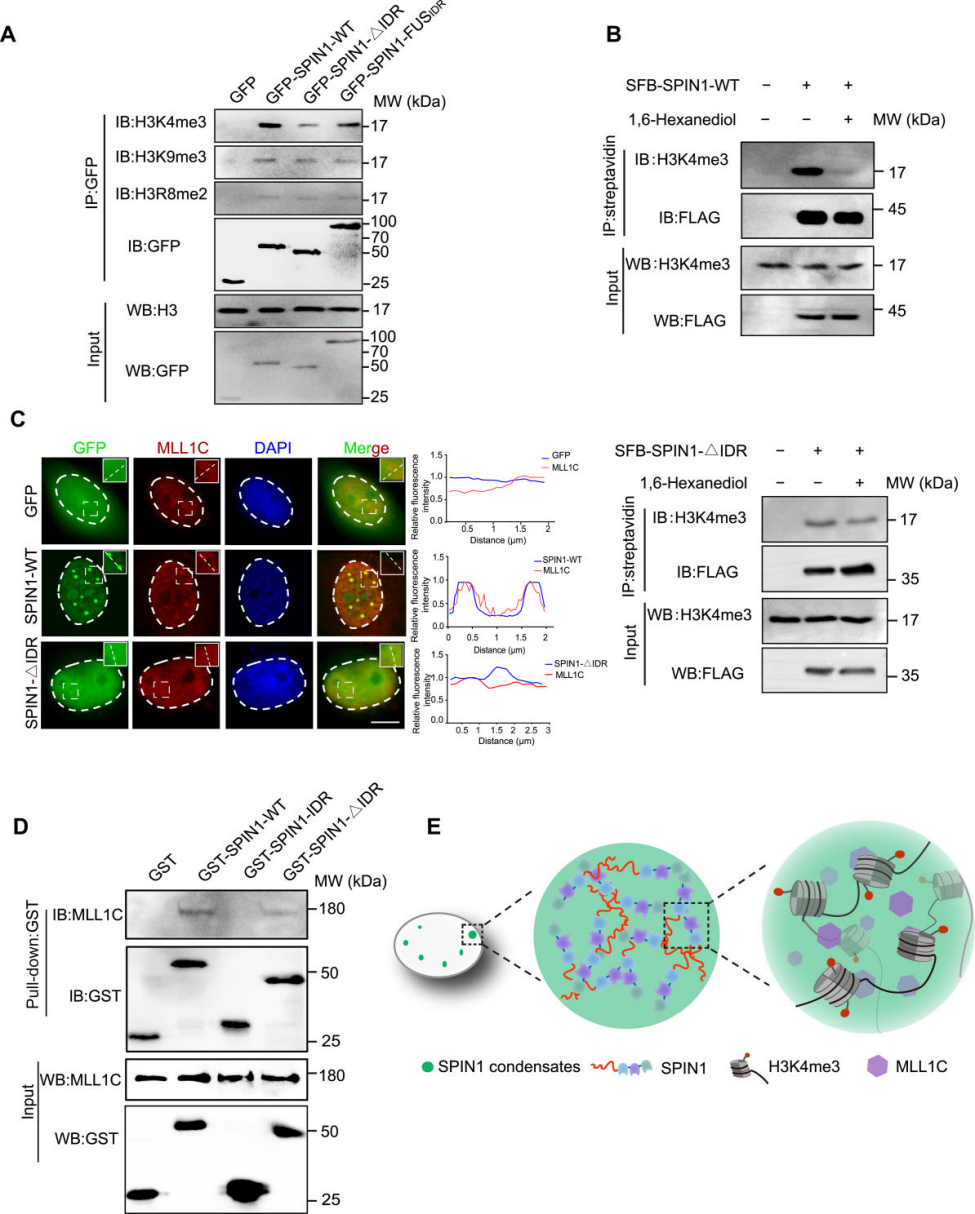
SPIN1-IDR promotes the recognition of H3K4me3 by SPIN1. (**A**) Loss of SPIN1-IDR compromises the binding of SPIN1 to H3K4me3 but does not affect the binding to H3K9me3 or H3R8me2. SGC7901 cells were transfected with GFP or the SPIN1 mutants. Then, the whole-cell lysates were immunoprecipitated with GFP antibody. (**B**) 1,6-Hexanediol impairs the binding of SPIN1 to H3K4me3. SGC7901 cells stably expressing SFB-SPIN1-WT (top) or SFB-SPIN1-∆IDR (bottom) were treated with or without 1% 1,6-hexanediol before being harvested for IP and western blotting. (**C**) MLL1C is partially recruited to the condensates formed by SPIN1. Left: co-staining images of SPIN1-WT or SPIN1-∆IDR with MLL1C. Scale bar, 5 μm. Right: statistical analysis of the fluorescence signals. (**D**) The interaction between GST-SPIN1 or the mutants and endogenous MLL1C. The recombinant proteins were pretreated with 10% (*w*/*v*) PEG5000 to facilitate the formation of condensates and then incubated with cell lysates. (**E**) A proposed model illustrating the regulatory role of SPIN1 condensation in facilitating the local enrichment of MLL1C and promoting the binding ability of SPIN1 to histone methylation.

SPIN1 is a histone methylation reader that specifically recognizes H3K4me3, H3K4me3R8me2, or H3K4me3K9me3 through its Tudor-like domains. However, the role of SPIN1-IDR in regulating histone methylation recognition remains elusive. By performing the co-immunoprecipitation (co-IP) assay, we found that SPIN1-∆IDR significantly compromised the binding ability of SPIN1 to H3K4me3 in SGC7901 cells (Figure 3A), consistent with the concept that condensates with reduced dynamicity can impair the molecular activity of a phase-separated gene regulator (Li et al., 2020c). However, SPIN1-∆IDR did not affect the binding ability of SPIN1 to H3R8me2 or H3K9me3. Interestingly, SPIN1-FUS_IDR_ was able to bind to H3K4me3 but not to the other two modifications (Figure 3A), suggesting that the IDR might facilitate binding with H3K4me3 through the formation of condensates. Consequently, we propose that the IDR serves as a region involved in regulating the function of SPIN1.

In SGC7901 cells ectopically expressing SPIN1-WT or SPIN1-∆IDR, 1,6-hexanediol treatment markedly decreased the interaction between SPIN1-WT and H3K4me3 but did not affect the binding of SPIN1-∆IDR with H3K4me3 (Figure 3B), suggesting that phase separation mediated by SPIN1-IDR could enhance the binding of SPIN1 with H3K4me3. We next studied how SPIN1 condensates regulate the recognition of histone modifications. The methylation of H3K4 is catalyzed by the histone methyltransferase MLL1 (Cao et al., 2014), and the MLL1 protein is proteolytically cleaved in the cell into N-terminal (MLL1N) and C-terminal (MLL1C) proteins. We observed that the endogenous MLL1C displayed a diffuse nuclear localization in cells transfected with the GFP vector or GFP-SPIN1-∆IDR, whereas a small amount of MLL1C co-localized with SPIN1-WT within cells transfected with SPIN1-WT (Figure 3C), indicating that only SPIN1-WT, but not SPIN1-∆IDR, could recruit MLL1C to the condensates. Thus, condensates formed by SPIN1 might selectively incorporate and enrich MLL1C in the same condensates to facilitate histone modification. However, there was no significant difference in the overall level of H3K4me3 between cells expressing SPIN1-WT and SPIN1-∆IDR (Supplementary Figure S3), suggesting that SPIN1 condensation may not directly enhance the global methylation activity of MLL1C. We then performed GST pull-down assay to confirm the interaction between SPIN1 and MLL1C. We found that loss of IDR significantly impaired the interaction between SPIN1 and MLL1C, but IDR alone did not directly bind to MLL1C (Figure 3D). These findings further indicate that SPIN1 condensation mediated by IDR has the capacity to compartmentalize and concentrate the specific histone methyltransferase (Figure 3E). Therefore, SPIN1 condensation facilitates local enrichment of the methyltransferase and promotes the histone methylation-binding ability of SPIN1, and SPIN1-IDR likely positively regulates SPIN1-mediated transcriptional activation by promoting the SPIN1 histone methylation-binding ability.

### SPIN1-IDR facilitates target gene activation and tumorigenesis *in vivo*

**Figure 4:**
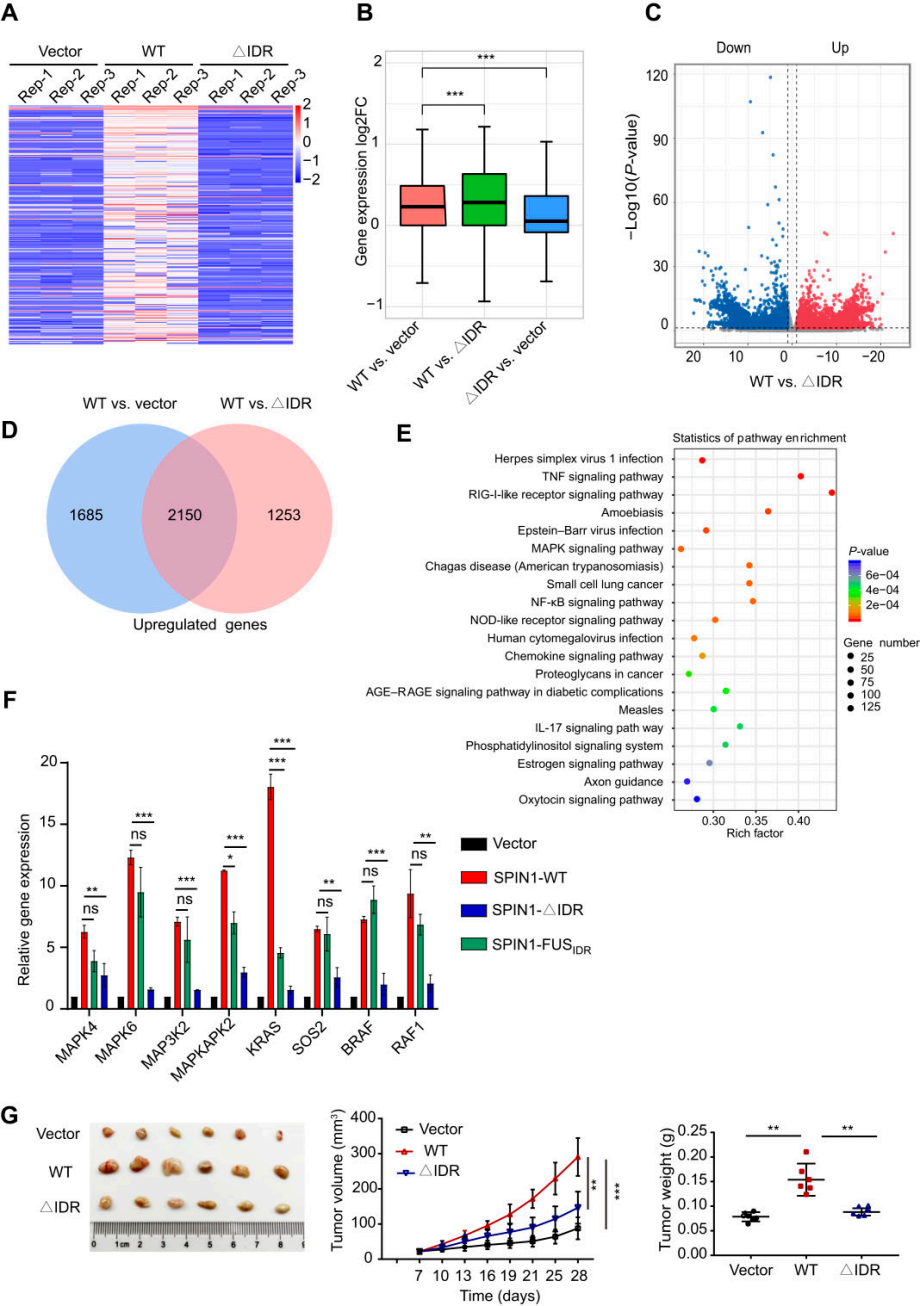
SPIN1-IDR regulates gene expression and tumor formation. (**A**) Heatmap of the top 300 upregulated genes in cells expressing SPIN1-WT compared with cells expressing the empty vector or SPIN1-∆IDR. (**B**) Box plots showing the relative expression of the top 300 genes in **A**. (**C**) A volcano plot showing the statistically upregulated (right) and downregulated (left) genes. (**D**) Venn diagrams showing the numbers of uniquely or commonly upregulated genes in SPIN1-WT vs. vector and SPIN1-WT vs. SPIN1-∆IDR. (**E**) KEGG enrichment analysis of the differentially expressed genes in cells expressing SPIN1-WT vs. SPIN1-∆IDR. (**F**) Relative mRNA levels of the indicated genes associated with the MAPK signaling pathway. Data from triplicate experiments are presented as mean ± SD. (**G**) SPIN1-WT but not SPIN1-∆IDR significantly facilitates xenograft tumor growth. Left: images of the xenograft tumors. Middle: curves of tumor volume. Right: tumor weight. *n* = 6/group.

To assess the role of IDR in regulating the expression of target genes, we performed transcriptome analysis by RNA sequencing (RNA-seq) with SGC7901 cells stably expressing SPIN1-WT or SPIN1-∆IDR. RNA-seq identified 3403 differentially expressed genes that were significantly upregulated in cells expressing SPIN1-WT (Figure 4A–D). Kyoto Encyclopedia of Genes and Genomes (KEGG) analysis of these data revealed that the top enriched pathways in cells overexpressing SPIN1-WT were the TNF signaling pathway, MAPK signaling pathway, NF-κB signaling pathway, etc. (Figure 4E). Previous studies indicated that SPIN1 directly enhances the expression of GDNF, an activator of the RET signaling pathway, to promote tumorigenesis (Franz et al., 2015). Activated RET primarily activates the RAS–MAPK and PI3K–AKT signaling pathways, which regulate cell proliferation and survival (Mulligan, 2014; Plaza-Menacho et al., 2014). We selected several differentially expressed genes in the MAPK signaling pathway and examined their expression levels in cells expressing SPIN1-WT or SPIN1-∆IDR by real-time quantitative polymerase chain reaction (RT-qPCR). Our results demonstrated that SPIN1-WT significantly increased the transcription of *MAPK4, MAPK6, MAP3K2, MAPKAPK2, KRAS, SOS2, BRAF*, and *RAF1*, all of which play important roles in regulating cell proliferation, while SPIN1-∆IDR did not significantly induce the gene expression but the SPIN1-FUS_IDR_ mutant enhanced the transcription of several genes, suggesting that IDR-mediated phase separation contributes to the regulation of gene expression (Figure 4F; Supplementary Figure S4B).

Accumulating reports have demonstrated the upregulation of SPIN1 in various cancer tissues, implicating its role in promoting cancer cell proliferation and tumorigenesis (Yuan et al., 2008; Zhang et al., 2008; Fang et al., 2018; Zhou et al., 2021). By analyzing cancer mutation data from the cBioPortal database, we found that the presence of deletions or missense mutations on SPIN1 in cancer cases correlates with a poor patient survival rate (Supplementary Figure S4A). Next, we examined the functional significance of SPIN1-IDR in gastric cancer cell proliferation and xenograft tumor growth in nude mice. Notably, compared to cells expressing SPIN1-WT, cells expressing SPIN1-∆IDR exhibited a reduced proliferation rate, while the SPIN1-FUS_IDR_ chimera successfully restored the cell proliferation capacity (Supplementary Figure S4B and C). In mice, the deletion of IDR significantly suppressed xenograft tumor growth without affecting body weight (Figure 4G; Supplementary Figure S4D). Collectively, our results establish the crucial role of SPIN1-IDR in target gene activation and tumorigenesis.

### SPIN1-IDR enhances the binding of SPIN1 to chromatin and the localization of SPIN1 to genes involved in the MAPK signaling pathway

**Figure 5:**
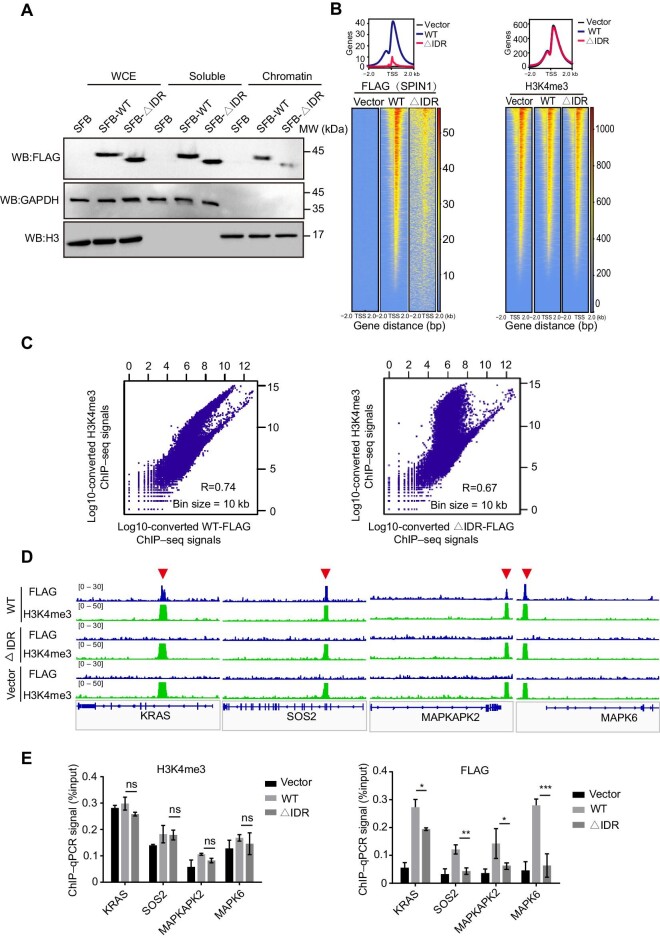
SPIN1-IDR occupies the promoter region of genes involved in the MAPK signaling pathway. (**A**) Loss of IDR significantly reduces the chromatin-binding ability of SPIN1. Whole-cell extracts (WCE), along with soluble and chromatin-bound SPIN1, were isolated from SGC7901 cells. (**B**) Heatmap (lower) and composite plot (upper) for SPIN1 or H3K4me3 peaks in the indicated cells. Each row represents a peak called for FLAG or H3K4me3 occupancy at gene loci ±2 kb around the TSS. (**C**) Scatterplots showing the correlation between the ChIP–seq signals of SPIN1-WT (left) or SPIN1-∆IDR (right) and H3K4me3. (**D**) Representative integrative genomics viewer browser tracks for the indicated genes associated with the MAPK signaling pathway. (**E**) ChIP–qPCR validations of the binding sites in **D** with anti-FLAG or anti-H3K4me3 antibodies.

As a transcription co-activator, SPIN1 has been reported to bind to chromatin (Wang et al., 2011; Bae et al., 2017a). Next, we performed chromatin fractionation to analyze the distribution of SPIN1-WT and SPIN1-∆IDR in the chromatin-bound and unbound fractions. The majority of SPIN1-WT but very few SPIN1-∆IDR was found in chromatin fractions, indicating that deletion of IDR largely dissociates SPIN1 from chromatin (Figure 5A). The major reason for the release of SPIN1-∆IDR from chromatin might be the impairment of the interaction between SPIN1 and H3K4me3 due to the loss of IDR.

Furthermore, we investigated the genome-wide chromatin association of SPIN1-WT or SPIN1-∆IDR by chromatin immunoprecipitation followed by high-throughput sequencing (ChIP–seq). ChIP–seq analysis revealed 18364 binding sites for SPIN1-WT, of which 62.33% were located at the promoter regions (Supplementary Figure S5A–C). SPIN1-∆IDR exhibited significantly less binding sites and weaker signals at promoter regions (Figure 5B; Supplementary Figure S5C). Of note, as a histone methylation reader, SPIN1 does not govern the localization of H3K4me3 sites. Therefore, the H3K4me3 occupancy sites within ±2 kb around the transcription start site (TSS) are similar in cells expressing SPIN1-WT and SPIN1-∆IDR (Figure 5B). SPIN1-WT signals considerably overlapped with H3K4me3 signals, which is associated with the transcriptional activation of nearby genes at multiple loci and suggests that both SPIN1 and H3K4me3 occupy the promoter regions of these genes. Importantly, these effects by SPIN1-WT tended to be diminished in cells expressing SPIN1-∆IDR (Figure 5C). Thus, the deletion of IDR impairs genome-binding ability of SPIN1.

Our ChIP–seq analysis revealed the presence of SPIN1 at the promoter of 11446 genes, with a significant co-localization between SPIN1 and H3K4me3. In contrast to previous studies that focused on SPIN1 promoting tumorigenesis through four tumor-associated signaling pathways (Wnt, PI3K/AKT, RET, and ribosomal subunit protein–MDM2–p53), our research identified a distinct and significant occupancy of SPIN1-WT at genomic regions associated with the MAPK signaling pathway. We observed unique peaks exclusively present in SPIN1-WT but not in SPIN1-∆IDR, which were enriched at the promoter regions for genes in the MAPK signaling pathway (Figure 5D; Supplementary Figure S5D). We also validated the enrichment of SPIN1 at gene regions associated with the MAPK signaling pathway by ChIP combined with qPCR (ChIP–qPCR) (Wu et al., 2020; Figure 5E; Supplementary Figure S5E). Collectively, these findings indicate that SPIN1-IDR not only facilitates the association of SPIN1 with chromatin but also enhances the localization of SPIN1 to genes involved in the MAPK signaling pathway, ultimately promoting cell proliferation and tumorigenesis.

## Discussion

Human SPIN1 was initially characterized as an H3K4me3 reader and recently reported to recognize bivalent H3K4me3R8me2 and H3K4me3K9me3 marks (Yang et al., 2012; Su et al., 2014; Du et al., 2021). SPIN1 is also involved in cell-cycle regulation, chromosome stability, multinucleated giant cell formation, cancer cell proliferation and metastasis, and abnormal tumor lipid metabolism (Yuan et al., 2008; Zhang et al., 2008; Franz et al., 2015). Here, we focus on the function of the previously unappreciated IDR of SPIN1. Our studies reveal that SPIN1 exhibits condensate properties in nucleus, and SPIN1-IDR is required for SPIN1 condensate formation, indicating that SPIN1-IDR plays a regulatory role in modulating the function of SPIN1. Furthermore, the condensate properties of SPIN1 are relevant to its recognition of histone methylation marks. Mechanistically, the phase separation of SPIN1 facilitates the enrichment of MLL1 within the condensates formed by SPIN1, resulting in an increase in local H3K4me3 levels. However, further studies are needed to establish a more definitive connection between the phase separation of SPIN1 and the modulation of local H3K4me3.

**Figure 6:**
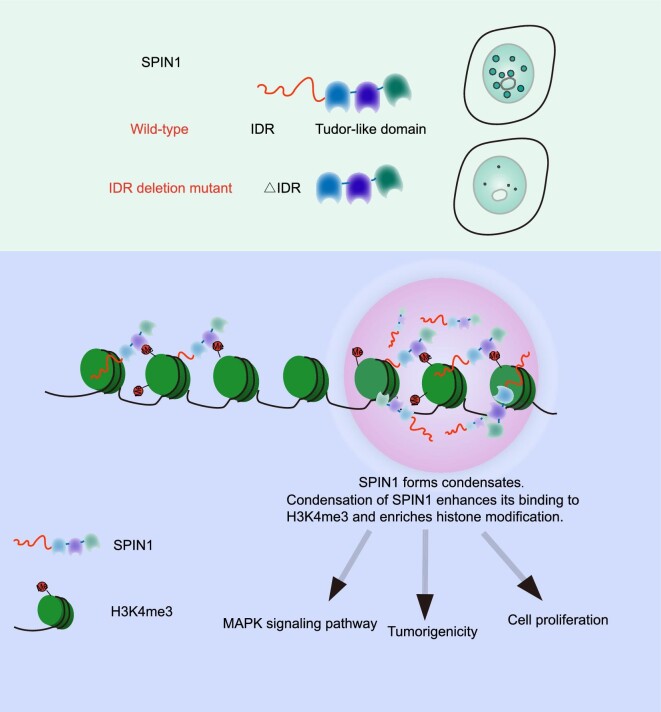
A proposed model illustrating that SPIN1 forms condensates via LLPS to facilitate the recognition of H3K4me3. SPIN1 forms phase-separated and liquid-like condensates through its N-terminal IDR. This process facilitates the binding of SPIN1 to H3K4me3 and activates tumorigenesis-related genes.

As a histone methylation reader and transcription co-activator, SPIN1 contributes to tumorigenesis by directly facilitating the expression of target genes within the MAPK signaling pathway. SPIN1-mediated transcriptional regulation of genes involved in the MAPK signaling pathway requires its binding to H3K4me3, which is facilitated by the phase separation properties of SPIN1-IDR. Thus, the phase separation properties of SPIN1 not only aid in the recognition of H3K4me3 and the recruitment of additional components on chromatin but also directly promote the expression of target genes involved in the MAPK signaling pathway (Figure 6). Taken together, the condensation of SPIN1 promotes efficient interactions among SPIN1, MLL1, and H3K4me3, spatially enhancing H3K4me3 recognition and transcriptional activation of target genes.

IDRs widespread in eukaryotes have emerged in recent years as key regulators of biomolecular condensates (Uversky, 2017; Chong and Mir, 2021). Accumulating evidence has shown that IDR-containing proteins are involved in a variety of essential biological processes (Ward et al., 2004; Wright and Dyson, 2015; Necci et al., 2016) and implicated in many diseases, such as Alzheimer's disease, cancer, amyotrophic lateral sclerosis, and Rett syndrome (Melo et al., 2016; Li et al., 2020a; Ahn et al., 2021; Shi et al., 2021), which makes them promising targets for drug discovery (Cheng et al., 2006). However, IDRs are historically understudied due to the flexible fold under physiological conditions and the difficulties in obtaining the exact structure and direct measurement of their dynamic behavior.

Here, we raise a question of how SPIN1-IDR regulates the chromatin reader function of SPIN1. We found that the droplet-like structure formed by SPIN1 in a phase-separated manner *in vitro* or *in vivo* depends on the N-terminus of SPIN1-IDR. In the nucleus, SPIN1 forms liquid-like condensates that can be dynamically fused, and the binding of SPIN1 to H3K4me3 is impaired when phase separation of SPIN1 is destroyed by drugs. Replacing the IDR of SPIN1 with FUS_IDR_ effectively restores the biological functions of SPIN1. A recent study also reported that different SPIN1 constructs (SPIN1_49–262_ and SPIN1_26–262_) exhibit varying binding affinity for the same SPIN1 inhibitor (Xiong et al., 2019), indicating a modulatory role of the N-terminus of SPIN1. Previous studies showed that the Tudor-like domains of SPIN1 can specifically bind to H3K4me3 in a ‘rigid mechanism’ way, while our study reveals that SPIN1 regulates gene expression by recognizing H3K4me3 in a ‘flexibility mechanism’ way. Our study has confirmed that SPIN1 partially enriches MLL1 into the liquid-like condensates and facilitates histone methylation recognition. However, further research utilizing genetic knockout strategies, such as CRISPR–Cas9 technology, to generate endogenously knockout clones of SPIN1-IDR is necessary to validate our findings in more physiological settings, as the overexpression-based assays and co-IP assays utilized in our study may have limitations. Altogether, SPIN1 condensation facilitates the recognition of histone modification marks by SPIN1, enabling the activation of target gene expression through forming a local structure, in which the histone modifications are enriched and its interaction with other factors are more efficiently orchestrated in a ‘rigid and flexible’ way. Previous studies also reported the potential of small-molecule inhibitors that target the ‘rigid’ Tudor-like 1 and Tudor-like 2 domains of SPIN1 in cancer treatment (Bae et al., 2017b; Xiong et al., 2019). Our study emphasizes the significance of the ‘flexible’ IDR of SPIN1 in regulating its functionality, and thus further research is needed to discover potent and selective inhibitors that disrupt phase separation properties mediated by SPIN1-IDR. In addition, the mechanism by which IDR promotes specific H3K4me3 binding through phase separation is complex and requires further investigation. For instance, the IDR of SPIN1 may have potential interaction partners or mediate other functions.

In summary, the IDR of SPIN1 promotes the formation of phase-separated liquid-like SPIN1 condensates *in vitro* and *in vivo*, and the SPIN1 condensates function as hubs where the transcription of target genes in the related signaling pathway is dynamically controlled. Thus, SPIN1-IDR is an indispensable region for the functions of SPIN1 and might also be a potential target for drug design. Our in-depth study on the ‘flexible mechanism’ of SPIN1 function not only reveals a novel mechanism for SPIN1 function but also provides a new perspective for drug design and development for cancer treatment, which is highly valuable in both academic research and biomedical application.

## Materials and methods

### Antibodies

The following antibodies were used for western blotting, IP, or immunofluorescence assays: anti-SPIN1 (Proteintech, #12105-1-AP), anti-PRA194 (Santa Cruz, #SC-48385), anti-H3K4me3 (Cell Signaling Technology, #9751), anti-H3K9me3 (Cell Signaling Technology, #13969), anti-H3R8me2 (NOVUS, #NB21-1062), anti-GST (ZSGB-BIO, #TA-03), anti-H3 (Cell Signaling Technology, #3638), anti-GAPDH (Cell Signaling Technology, #2118), anti-FLAG (Sigma, #F1804), anti-GFP (Proteintech, #50430-2-AP), anti-GFP (Proteintech, #66002-1-Ig), and anti-MLL1 (Cell Signaling Technology, #14197). Horseradish peroxidase (HRP)-conjugated secondary antibodies were purchased from Cell Signaling Technology (#7076 and #7074). Fluorescent secondary antibodies were purchased from Invitrogen (#A-11034, #A-11012, and #A-11032).

### Cell fractionation and western blotting

Cells were lysed with NETN 100 lysis buffer (20 mM Tris–HCl, pH 8.0, 100 mM NaCl, 1 mM EDTA, and 1% NP-40) on ice for 30 min. After centrifugation, the supernatant was harvested and used as the soluble fraction. The insoluble pellets were collected, washed with ice-cold phosphate-buffered saline (PBS), and incubated with NETN 300 buffer (20 mM Tris–HCl, pH 8.0, 300 mM NaCl, and 1% NP-40) containing 0.5 U/μl benzonase and 5 mM MgCl_2_ on ice. After centrifugation, the supernatant was collected as the chromatin fraction. For whole-cell extracts, cells were lysed directly in NETN 300 containing 0.5 U/μl benzonase. Then, 5× protein loading buffer (25 mM Tris–HCl, pH 6.8, 10% sodium dodecyl sulfate (SDS), 5 mg/ml bromophenol blue, 50% glycerin, and 10% β-mercaptoethanol) was added to the samples, which were subsequently denatured at 98°C for 5 min. Denatured proteins were loaded on SDS–polyacrylamide gel electrophoresis (PAGE) and transferred to polyvinylidene fluoride membranes (Merck Millipore, #IPVH00010) using the Bio-Rad Mini Trans-Blot^®^ Electrophoretic Transfer System. After blocking with 5% non-fat milk in 20 mM Tris–HCl (pH 8.0) containing 150 mM NaCl and 0.05% Tween-20 (TBST) for 40 min, the membranes were incubated with primary antibodies overnight. After washing three times with TBST, the membranes were incubated with HRP-conjugated secondary antibodies for 1 h at room temperature. Finally, the protein bands were visualized with an enhanced chemiluminescence detection kit (Thermo Fisher, #34577) and a ChemiDoc MP XRS+ Imaging System (Bio-Rad).

### Protein expression and purification

Both full-length SPIN1 and the IDR deletion mutant were expressed in *Escherichia coli* BL21 (DE3) cells. The proteins were tagged with N-terminal MBP-6×His or MBP-6×His-TEV-EGFP, as previously described (Wang et al., 2018; Du et al., 2021). In brief, the bacteria were cultured at 37°C until the density reached an OD_600_ of 0.8–1.0. Then, isopropyl β-d-1-thiogalactopyranoside was added to induce protein expression at 18°C overnight. Next, the cells were resuspended in lysis buffer (20 mM Tris–HCl, pH 8.0, and 500 mM NaCl) and lysed by sonication. After centrifugation (30000× *g*, 50 min), the supernatants were loaded onto a Ni-chelating Sepharose column (GE Healthcare). The soluble protein was eluted with elution buffer (20 mM Tris–HCl, pH 8.0, 500 mM NaCl, and 200 mM imidazole). Then, the N-terminal MBP tag was removed by TEV protease treatment. Untagged proteins were further purified by ion-exchange column Source 15Q (GE Healthcare) and Superdex 75 column (GE Healthcare). The SPIN1 mutant proteins were purified using the same protocol. The purified proteins were examined by SDS–PAGE, followed by Coomassie blue staining.

### Immunofluorescence staining

Cells were seeded into 6-well plates and grown overnight. After treatment with 0.1% Triton X-100 for 5 min at room temperature, the cells were fixed with 4% paraformaldehyde for 10 min and blocked with 8% goat serum for 30 min. The cells were then incubated with primary antibody diluted in 8% goat serum overnight at 4°C. After washing three times with PBS, the cells were incubated with secondary antibody diluted in 8% goat serum for 40 min. After washing with PBS, the nuclei were stained with 4′,6-diamidino-2-phenylindole (DAPI) for 5 min. The cells were mounted onto glass slides with anti-fade reagent (SouthernBiotech, #0100-01) and imaged by using a fluorescence microscope (Olympus, Fluoview FV3000).

### Computational prediction of disordered regions and analyses of protein structure

Computational prediction of disordered regions of SPIN1 was performed using IUPred3 (http://iupred.elte.hu/). The protein structure of full-length SPIN1 was analyzed by AlphaFold (https://alphafold.com/).

### FRAP assay

The FRAP assay was conducted using the FRAP module of the Olympus confocal microscopy system. GFP-tagged SPIN1-WT or SPIN1-∆IDR was bleached using a 488-nm laser with 80% power at a circular region of interest. After photobleaching, time-lapse images were captured every 5 sec for ∼1 min using a Fluoview FV3000 confocal laser scanning microscope (Olympus). ImageJ was used for quantification and GraphPad Prism was used for data analysis.

### *In vitro* phase separation assay

Briefly, purified fluorescent protein-fused SPIN1 proteins were diluted to a final concentration of 10 μM in a buffer containing 20 mM Tris–HCl (pH 8.0), 1 mM dithiothreitol (DTT), 150 mM NaCl, and 10% PEG5000. Subsequently, 5 μl of each sample was pipetted onto a glass dish. Fluorescent and differential interference contrast (DIC) microscopy images were captured using an Olympus confocal microscope.

The purified SPIN1-WT protein was diluted to a range of 1–25 μM in ‘condensation buffer’ (20 mM Tris–HCl, pH 8.0, 1 mM DTT, 75–300 mM NaCl, and 10% PEG5000) at 25°C for 10 min. The sample was pipetted onto a glass dish and DIC microscopy images were captured using an Olympus confocal microscope.

### ChIP–seq

Raw sequencing data were filtered out of the adaptor and low-quality reads by Trimmomatic (version 0.36). The clean reads were mapped to the reference genome of *Homo sapiens* from ftp://ftp.ensembl.org/pub/release-96/fasta/homo_sapiens/dna/ using STAR software (version 2.5.3a). The RSeQC (version 2.6) was used for read distribution analysis and MACS2 software (version 2.1.1) was used for peak calling. Besides, the peak annotation and peak distribution were further analyzed by bedtools (version 2.25.0). Then, both Gene Ontology (GO) analysis and KEGG enrichment analysis were implemented by KOBAS software (version 2.1.1). ChIP–seq was conducted by LC-Bio Technology Co., Ltd.

### RNA-seq

Total RNA was purified from SGC7901 cells using TRIzol reagent (Thermo Fisher, #15596018). The libraries were sequenced on Illumina Novaseq^™^ 6000 (LC-Bio Technology Co., Ltd) obtaining 2 × 150-bp paired-end reads. The reads were mapped to the human genome (hg38) using the HISAT2 (https://daehwankimlab.github.io/hisat2/, version hisat2-2.0.4) package. GO terms and KEGG pathways were analyzed to meet this condition with |NES| > 1, NOM *p*-val < 0.05, FDR *q*-val < 0.25.

### Statistical analysis

Statistical analysis was performed using GraphPad Prism 7.0. *P*-values were analyzed by Fisher's exact test or two-sided Student's *t*-test. Data are plotted with error bars representing the standard error of the mean (SEM) or standard deviation (SD). Statistical significance levels are denoted as follows: *P < 0.05;**P < 0.01; ***P < 0.001; ns, not significant.

### Data availability

The high-throughput sequencing data, including RNA-seq and ChIP–seq data, have been deposited in the Gene Expression Omnibus database (http://www.ncbi.nlm.nih.gov/geo/) with the accession number GSE215400. Cancer mutation data are available from the cBioPortal database (http://www.cbioportal.org/). Source data are provided with this paper.

## Supplementary Material

mjae024_Supplemental_File

## Notes

**Author contributions:** C.W. designed the experiments; Y.W. performed most of the experiments with the assistance of Y.C., Z.L., and Y.J.; Y.W. conducted the bioinformatic analyses under the guidance of Z.Y. and C.W.; Y.W., M.L., Y.Z., and J.W. performed the animal experiments and purified the proteins; C.W., R.X., and Z.Y. analyzed the data; and C.W. wrote the manuscript. All the authors read and approved the final manuscript.
