# Supplementary material for: Phase separation of SPIN1 through its IDR facilitates histone methylation readout and tumorigenesis
Source: J Mol Cell Biol. 2024 May 22;16(6):mjae024. doi: 10.1093/jmcb/mjae024 (PMC11630302; doi:10.1093/jmcb/mjae024)
Supplement: mjae024_Supplemental_File [file mjae024_supplemental_file.pdf]

Materials and methods

Plasmids

Human SPIN1 full length and △IDR were cloned into SFB vector (S-3×FLAG-SBP-tagged at the N-terminus), pEGFP-C1 (GFP-tagged at the N-terminus) or pET28b-MBP (MBP-6×His-taged at the N-terminus). The FUS_{IDR}-SPIN1, TDP-43_{IDR}-SPIN1, $SPIN1_{IDR}$-SPIN4, $SPIN4_{IDR}$-SPIN1, and the deletion mutations of SPIN1 were cloned into pEGFP-C1 vector (GFP-tagged at the N-terminus). pEGFP-SPIN1-WT or pEGFP-SPIN1-△IDR was used as a template to generate MBP-GFP-SPIN1-WT and MBP-GFP-SPIN1-△IDR expression vectors.

Cell culture and stable cell lines

U2OS or SGC7901 cells were cultured in DMEM medium with 10% fetal bovine serum, penicillin (100U/ml), streptomycin (100 g/ml) and cultivated at 37 °C in 5% CO_2 (v/v). To generate overexpression cell lines, SGC7901 cells were transfected with SFB-puro vector containing either SPIN1-WT or SPIN1-△IDR. Transfected cells were plated at a low density in 2.0 μg/ml puromycin. Individual clones were isolated and validated by western blotting using anti-FLAG antibody.

Immunoprecipitation

Cells were lysed with NETN 300 lysis buffer (20 mM Tris-HCl, pH 8.0, 300 mM NaCl, 1 mM EDTA and 1% NP-40). After centrifugation at 15,000g, 4℃ for 10 minutes, the supernatant was incubated with High Capacity Streptavidin Agarose (Thermo Fisher Scientific, #20359) for 2 hours at 4℃. Then, beads were collected by centrifugation at 1000g for 1 minute and washed with NETN 100 lysis buffer (20 mM Tris-HCl, pH 8.0, 100 mM NaCl, 1 mM EDTA and 1% NP-40). Bead-bound proteins were resolved by SDS–PAGE and detected by Western blot.

RT-qPCR

Total RNA was isolated from cells using TRIzol reagent (Invitrogen) and cDNA was reverse transcribed with random primer by using a Superscript III First Strand Synthesis Kit (Invitrogen, #18080051). RT-qPCR was performed with TB Green® Fast qPCR Mix (Takara，# RR430A) and the details of all primers used were provided in Supplementary Table S1. Gene expressions were calculated with comparative C_T

method using GAPDH as inner control.

ChIP–qPCR

ChIP assays were performed as described Previously(Wu et al., 2020). Briefly, cells were cross-linked with 1% formaldehyde at room temperature for 10 minutes. After centrifugation, the cell pellets were resuspended in SDS buffer containing 1% SDS, 10 mM EDTA, 50 mM Tris–HCl (pH 8.0), supplemented with protease-inhibitor cocktail (sigma, #P8340). The DNA was fragmented by sonication. After centrifugation, the supernatant was diluted with buffer (20 mM Tris–HCl (pH 8.0), 150 mM NaCl, 2 mM EDTA, and 1% Triton X-100). Protein A/G agarose containing salmon sperm DNA (Solarbio, #H1060) was used to clear the diluted chromatin. The lysates were incubated with 4 μg indicated antibodies or control IgG at 4 °C with rotation overnight. Immune complexes were pulled down with agarose beads for 2 hours. Beads were centrifuged at 2000× *g* for 1 minute after washing with following buffers: once in low salt buffer (20 mM Tris–HCl (pH 8.0), 150 mM NaCl, 1% SDS, 2 mM EDTA, 1% Triton X-100), once in high salt buffer(20 mM Tris–HCl (pH 8.0), 500 mM NaCl, 1% SDS, 2 mM EDTA, 1% Triton X-100),once in LiCl wash buffer(10 mM Tris–HCl (pH 8.0), 1% deoxycholic acid, 1 mM EDTA, 1% NP-40, and 0.25 M LiCl) and twice with Tris-EDTA buffer. DNA was eluted from beads with elution buffer (100 mM NaHCO3 and 1% SDS) at 65°C followed by treatment with 50 μg/ml RNase A (Solarbio, #R1030) and 100 μg/ml protease K (Merck, #1245680100). Then, DNA was purified by phenol–chloroform–ethanol. All precipitated DNA was quantified using RT-qPCR. Data are presented as the percentage of input. All ChIP experiments were performed at least three times. Primer sequences are listed in Supplementary Table S1.

Cell Counting Kit-8 (CCK-8) assay

Indicated cells were inoculated at a density of 2×10^3 cells per well in 96-well plates for 0, 12, 24, 36, and 48 h. After adding 10 μl of the CCK-8 reagent and incubated at 37 °C for 1 h, the absorbance values of treated cells were detected at 450 nm.

***In vivo* tumorigenicity assay**

Male BALB/c nude mice (4–5 weeks of age) were purchased from Weitong Lihua

Experimental Animal Technology Co. Ltd. 2×10^6 SGC7901 cells stably expressing SPIN1-WT or SPIN1-△IDR were washed and resuspended in 100 μl PBS. Cells were injected into male NSG mice, respectively. Tumor size was measured in the two longest dimensions using a Vernier caliper. Tumor volume (V) was calculated with the formula V=1/2 length×width×width. Four weeks after transplantation, the mice were euthanized to collect tumors. All animal experiments were approved by the Institutional Animal Care and Use Committee at Hebei University.

Figure S1

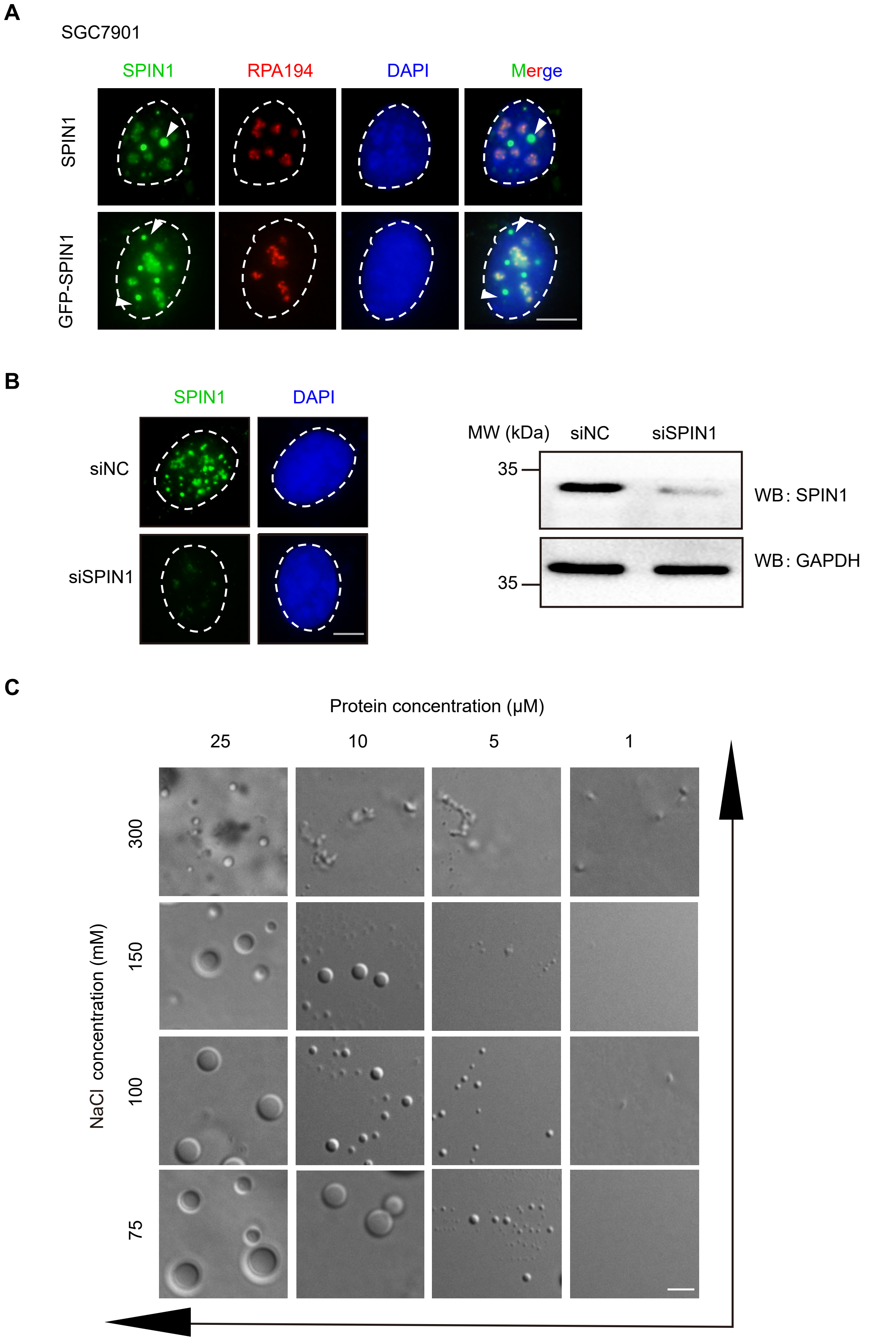
A
SGC7901
SPIN1
RPA194
DAPI
Merge
SPIN1
GFP-SPIN1
B
SPIN1
DAPI
siNC
siSPIN1
MW (kDa)
siNC
siSPIN1
35
WB : SPIN1
WB : GAPDH
35
C
Protein concentration (μM)
25
10
5
1
NaCl concentration (mM)
300
150
100
75

Supplementary Figure legends

Supplementary Figure S1. SPIN1 can form droplets both *in vivo* and *in vitro*.

(A) The representative images of the co-immunostaining of endogenous SPIN1 (top) or exogenous GFP-tagged SPIN1 (bottom) with the nucleolus marker RPA194 in SGC7901 cells. Scale bars,5μm. **(B)** The droplets disappeared in SPIN1 knocked down cells. Shown on the left are the fluorescence microscopy images of the SPIN1 in U2OS cells, which is transfected with siNC or siSPIN1. Scale bar,5μm. Shown on the right are immunoblotting showing the expression of SPIN1. **(C)** The images of SPIN1 protein at concentrations ranging from 1μm to 25μm in 20mM Tris (pH 8.0),10% (w/v) PEG5000 and sodium chloride (ranging from 75~300 mM). Scale bar,10μm.

Figure S2

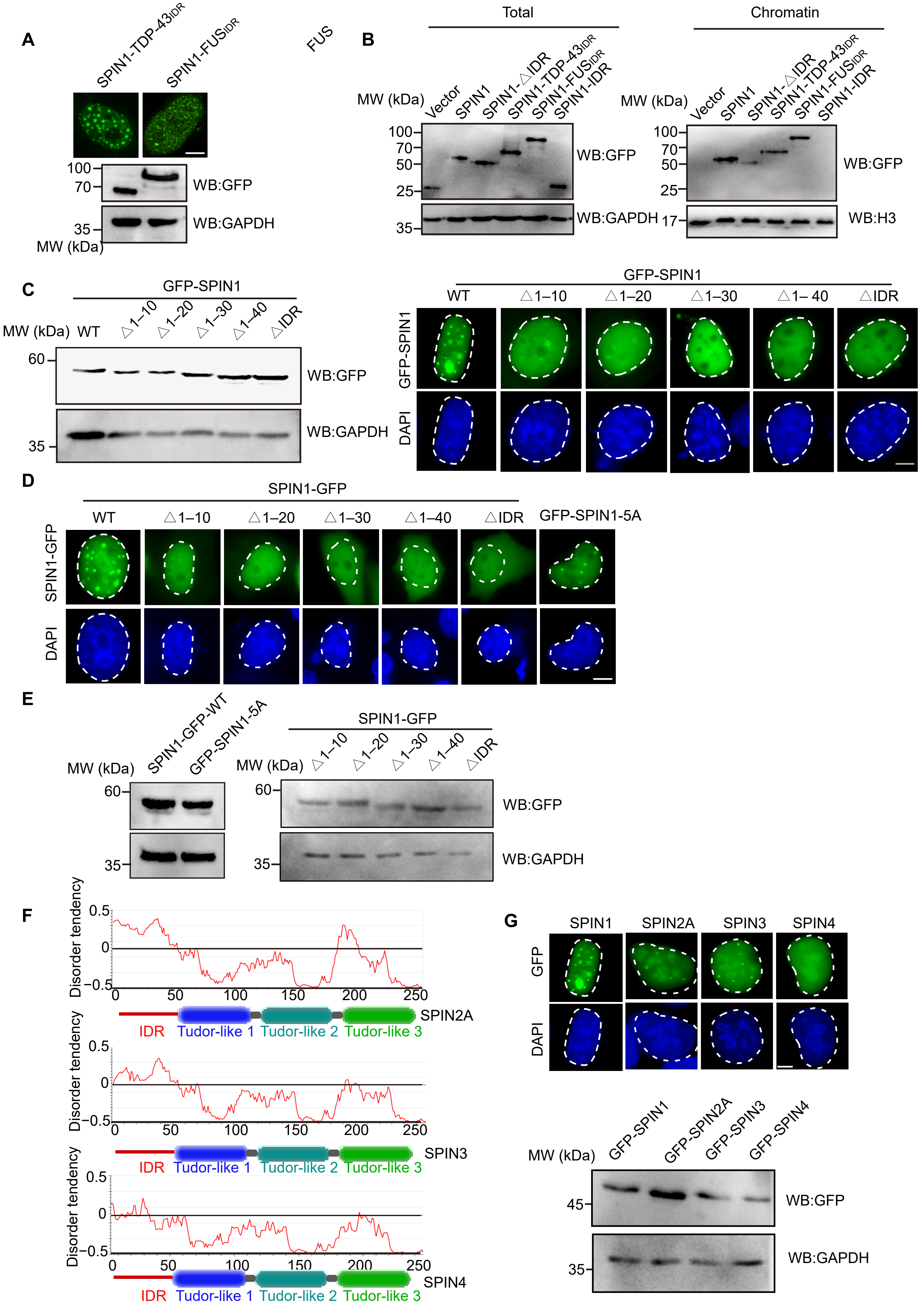

A
SPIN1-TDP-43IDR
SPIN1-FUSIDR
FUS
WB:GFP
WB:GAPDH
MW (kDa)
B
Total
Chromatin
MW (kDa)
Vector
SPIN1
SPIN1-△IDR
SPIN1-TDP-43IDR
SPIN1-FUSIDR
SPIN1-IDR
WB:GFP
WB:GAPDH
WB:H3
C
GFP-SPIN1
WT
△1–10
△1–20
△1–30
△1–40
△IDR
WB:GFP
WB:GAPDH
GFP-SPIN1
DAPI
D
SPIN1-GFP
GFP-SPIN1-5A
DAPI
E
SPIN1-GFP-WT
GFP-SPIN1-5A
SPIN1-GFP
WB:GFP
WB:GAPDH
F
Disorder tendency
SPIN2A
SPIN3
SPIN4
IDR
Tudor-like 1
Tudor-like 2
Tudor-like 3
G
SPIN1
SPIN2A
SPIN3
SPIN4
GFP
DAPI
GFP-SPIN1
GFP-SPIN2A
GFP-SPIN3
GFP-SPIN4
WB:GFP
WB:GAPDH

Figure S2

H

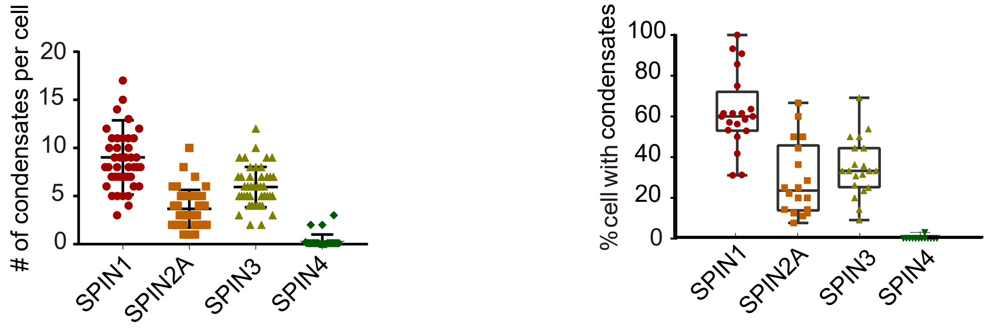

I

1 10 20 30 40 50

SPIN1 MKTPF GKTPG QR SRA DAGHA GVSAN MMKKR TSHKK HRS SV GPSKP VSQPR

SPIN2A MKTPN AQEAE GQQTR AAAGR ATG SA NM TKK KVSQK KQRGR PSSQP R

SPIN3 MKTPF GKAAA GQRSR TGAGH GSV SV TM I KR KAAHK KHRSR PTSQP R

SPIN4 MS PPT VPPMG VDGVS AYLMK KRHTH RKQ RR KP T FL TR

■ Non-Polar amino acids

_Polar amino acids (■ : Basic■ : Acidic■ : Neutral)

J

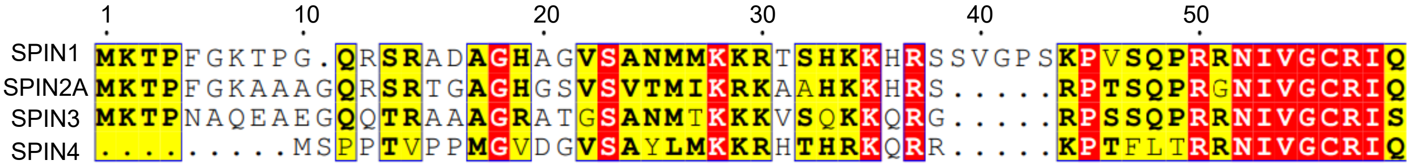

K

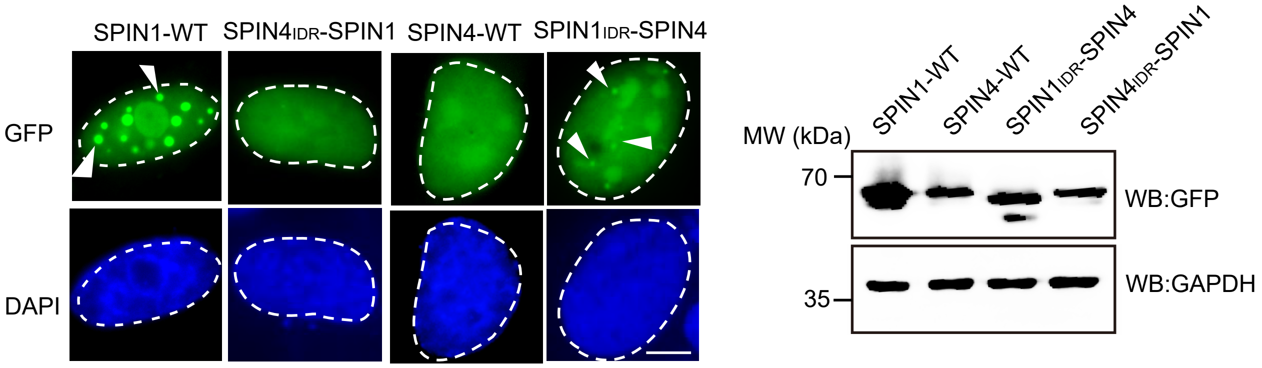

Supplementary Figure S2. The IDR of SPIN1 is essential for the droplet formation.

(A) The SPIN1 chimeras restore the nuclear droplet formation. Shown on the top are the representative images of the condensates formed by indicated EGFP tagged SPIN1 chimeric constructs (SPIN1-TDP-43$_{IDR}$ and SPIN1-FUS$_{IDR}$) in U2OS cells. Shown on the bottom are expressions of the indicated chimeras. **(B)** The SPIN1 chimera restores the ability of chromatin binding of SPIN1. Total cell lysates and chromatin-bound fractions extracted from cells expressing SPIN1, SPIN1-IDR, SPIN1-△IDR or SPIN1 chimeras were subjected to Western blotting and detected with the indicated antibodies. **(C)** The fluorescence microscopy images of U2OS cells transfected with indicated SPIN1 constructs fused to EGFP at the N-terminus. Shown on the left is the western blot showing the expression of SPIN1 mutants with partially deleted IDR. Shown on the right is the microscopy image of the indicated GFP-SPIN1 mutants in U2OS cells. Scale bar,5μm. **(D)** The fluorescence microscopy images of U2OS cells transfected with indicated SPIN1 constructs fused to EGFP at the C-terminus. A series of deletion mutations of SPIN1 were transfected into U2OS cells and the droplet formations were examined by immunofluorescence assay. Scale bars,5μm. **(E)** The expressions of SPIN1 mutants were examined by western blotting. **(F)** The disorder tendency of the protein sequences of SPIN2A, SPIN3 and SPIN4 analyzed by IUPred3. **(G)**The members of SPIN-family except for SPIN4 can form condensates *in vivo*. GFP-SPIN1, GFP-SPIN2A, GFP-SPIN3 or GFP-SPIN4 was transiently transfected into U2OS cells and the condensates formed by them were determined by fluorescent microscope. Show on the top is the representative fluorescence images. Scale bars,5μm. Show on the bottom is the protein expressions of the members of SPIN-family analyzed by Western blotting. **(H)**Quantification of the condensate numbers per cell and the percent of cells with condensate shown in G. Data are presented as mean ± s.d. n ≥50. **(I)** The amino acid composition of IDR region in SPIN families was analyzed. **(J)** Sequence alignment of the members of SPIN-family generated using ClustalW and displayed using ESPript. **(K)** The fluorescence microscopy images of U2OS cells transfected with GFP-SPIN1, SPIN1$_{IDR}$-SPIN4, GFP-SPIN4 or SPIN4$_{IDR}$-SPIN1 constructs. The formation of condensates by SPIN1 mutants were examined using fluorescent microscope. Show on the left is the representative fluorescence images. Scale bars,5μm. Show on the right is the protein expressions of the mutations analyzed by Western blotting.

Figure S3

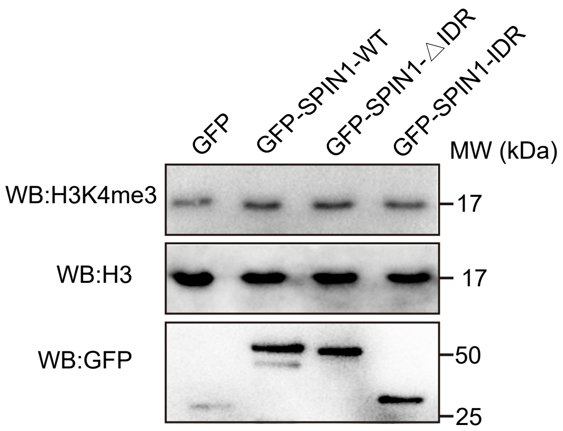
GFP
GFP-SPIN1-WT
GFP-SPIN1-△IDR
GFP-SPIN1-IDR
MW (kDa)
WB:H3K4me3
17
WB:H3
17
WB:GFP
50
25

Supplementary Figure S3. The overall level of H3K4me3 in cells expressing SPIN1-WT or deletion mutants shows no significant difference.

SPIN1-WT and deletion mutants were transfected into SGC7901 cells, and the total level of H3K4me3 were examined by Western blotting using indicated antibody.

Figure S4

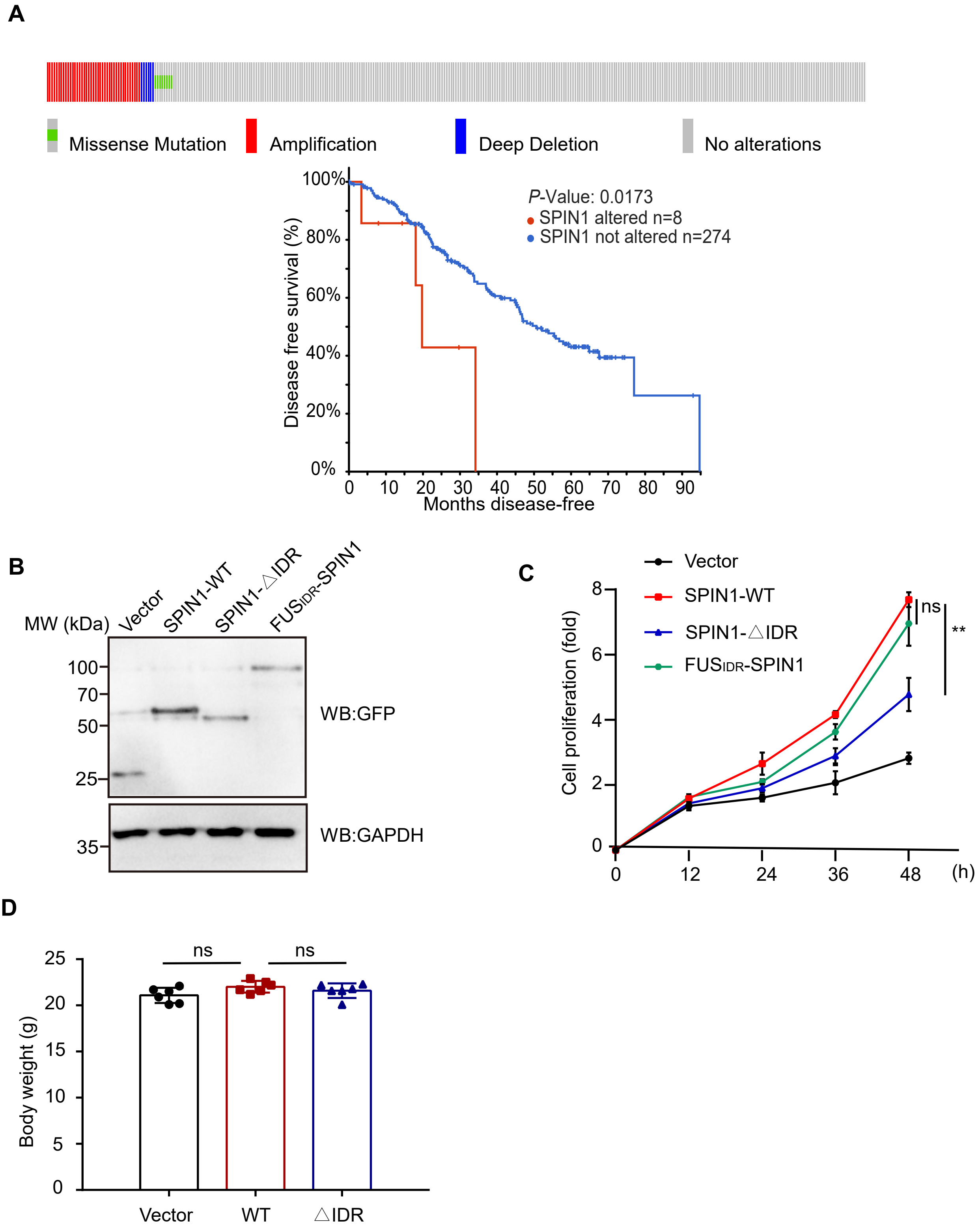
A
Missense Mutation
Amplification
Deep Deletion
No alterations
P-Value: 0.0173
SPIN1 altered n=8
SPIN1 not altered n=274
Disease free survival (%)
100%
80%
60%
40%
20%
0%
0
10
20
30
40
50
60
70
80
90
Months disease-free
B
MW (kDa)
Vector
SPIN1-WT
SPIN1-△IDR
FUSIDR-SPIN1
100
70
50
25
35
WB:GFP
WB:GAPDH
C
Vector
SPIN1-WT
SPIN1-△IDR
FUSIDR-SPIN1
Cell proliferation (fold)
8
6
4
2
0
0
12
24
36
48
(h)
ns
**
D
Body weight (g)
25
20
15
10
5
0
ns
ns
Vector
WT
△IDR

Supplementary Figure S4. IDR of SPIN1 potentiates cell proliferation.

(A) Mutations of SPIN1 in cancer are correlated with poorer patient survival. Top: each box represents a patient sample. Bottom: The survival curves showed that patients with altered SPIN1 had a poorer disease-free survival rate. **(B)** The protein expressions of the SPIN1-WT and the mutants were analyzed by Western blotting.**(C)** Loss of IDR impairs SPIN1's ability of promoting cell proliferation. Cell proliferation was measured by CCK8 assay in SGC7901 cells expressing SPIN-WT, SPIN1-△IDR or FUS_{IDR}-SPIN1. The data was shown as mean ± s.d. **(D)**The weight of the mice at the time of xenografts tumor being harvested. Mean ± s.d.

Figure S5

A

Cell sample	Epitope for ChIP	Total mapped #
Vector (FLAG-taged)	Input	21,451,626
	FLAG ChIP	19,555,052
SPIN1-WT (FLAG-taged)	Input	18,498,690
	FLAG ChIP	19,378,558
SPIN1-△IDR (FLAG-taged)	Input	19,087,348
	FLAG ChIP	17,660,550
Vector (FLAG-taged)	Input	97,398,698
	H3K4me3 ChIP	100,032,350
SPIN1-WT (FLAG-taged)	Input	83,853,314
	H3K4me3 ChIP	78,581,714
SPIN1-△IDR (FLAG-taged)	Input	72,304,904
	H3K4me3 ChIP	81,421,408

B

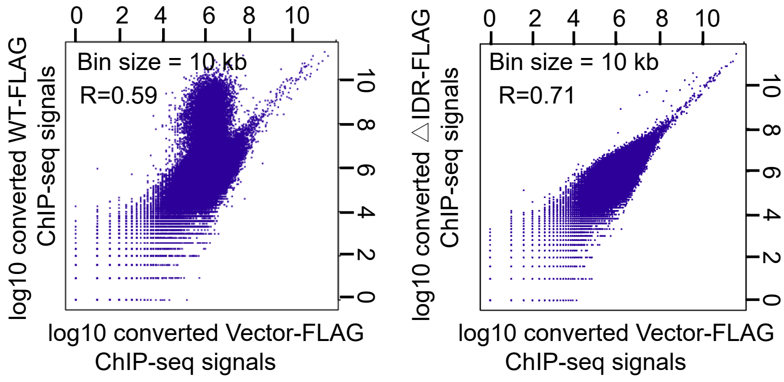

C

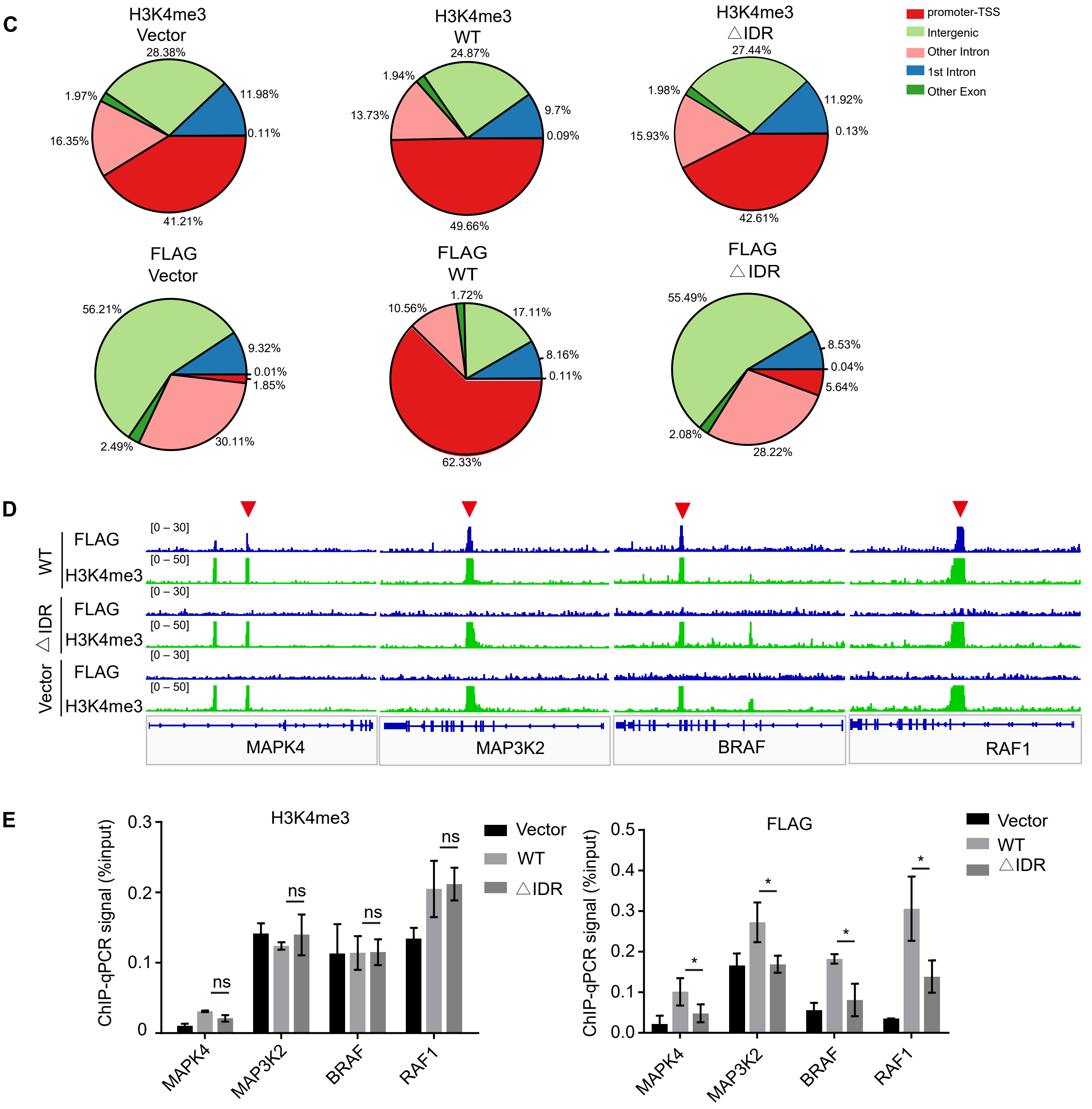

Supplementary Figure S5. ChIP-seq reveals the binding patterns of SPIN1-WT or SPIN1-△IDR to genome.

(A) Summary of the counts of ChIP–seq read tags for the indicated samples. **(B)**The binding signals of SPIN1-△IDR have a higher correlation with those of vector compared to SPIN1-WT. Scatterplots showing the correlation of global SPIN1-WT or SPIN1-△IDR ChIP-seq signals using FLAG antibodies. Coefficient of determination (R) is determined by Pearson correlation. **(C)** Pie chart showing the distribution of indicated annotation feature in SGC7901 cells stably expressing empty vector, SFB-SPIN1-WT or SFB-SPIN1-△IDR. **(D)** Integrative Genomics Viewer (IGV) browser tracks of the indicated genes associated with MAPK signaling pathway. **(E)** ChIP-qPCR validation of the binding sites in D. Data are presented as mean ± s.d. P value was evaluated by Student's t test.

primers

SPIN1-N1-GFPC1-*Sal* I-5	ACGC*GTCGAC* ATGAAGACCCCATTCGGAAAGAC
SPIN1-C262-GFPC1-*Bam* HI	CG*GGATCC* CTAGGATGTTTTCACCAAATCG
SPIN2A-GFPN1-*Eco* R1-5	CG*GAATTC* ATGAAGACCCCCAACGCACAG
SPIN2A-GFPN1-*Bam* H1-3	CGGGATCCTTAGGACTTTTTCACCAAATCG
SPIN3-GFPN1-*Xho* 1-5	CCG*CTCGAG* ATGAAGACCCCGTTTGGAAAG
SPIN3-GFPC1-*Bam* H1-3	CG*GGATCC* CTAAGATGTTTTTACCAAATCG
SPIN4-GFPN1-*Eco* R1-5	CG*GAATTC* TATGTCTCCTCCAACCGTGCCTCC
SPIN4-GFPC1-*Bam* H1-3	CG*GGATCC* TTAAGGAGTTTTCACCAAACCATAG
SPIN-N51-GFPC1-*Sal* I-5	ACGC*GTCGAC* CGGAACATCGTAGGCTGCAG
SPIN1-N1-*Eco* R1-SFB-5	CG*GAATTC* ATGAAGACCCCATTCGGAAAGAC
SPIN1-C262-*Bam* H1-SFB-3	CG*GGATCC* CTAGGATGTTTTCACCAAATCG
SPIN1-N51-*Eco* R1-SFB-5	CG*GAATTC* CGGAACATCGTAGGCTGCAG
SPIN1-GFP-C1-3	GCTTGAGCTCGAGATCTGAGTC
SPIN1-△1-10-N11-5	CAGCGGTCCAGAGCTGATG
SPIN1-△1-20-N21-5	GGAGTATCTGCCAACATGATG
SPIN1-△1-30-N31-5	ACATCCCACAAAAAACATCG
SPIN1-△1-40-N41-5	GGTCCGAGCAAACCTGTTTC
SPIN1-GFP-N1-3	CTGAGTCCGGTAGCGCTAG
SPIN1-N1-*Bam* H1-MBP-5	CG*GGATCC* ATGAAGACCCCATTCGGAAAGAC
SPIN1-C262-*Xho* I-MBP-3	CCG*CTCGAG* CTAGGATGTTTTCACCAAATCG
SPIN1-F-1-50	CGAATTCTGCAGTCGACATGAAGACCCCATTCGGAAAG
SPIN1-R-50	CAGCCCACGATGTTCCTCCGGGGCTGGGAAAC
SPIN4-38-249-R	CTTTCCGAATGGGGTCTTCATGTCGACTGCAGAATTCG
SPIN4-38-249-F	GTTTCCCAGCCCCGGAGGAACATCGTGGGCTG
SPIN4-IDR-F	CGAATTCTGCAGTCGACATGTCTCCTCCAACCGTG
SPIN4 IDR-R	CAGCCTACGATGTTCCGACGAGTGAGGAAAGTGGG

ChIP-QPCR primers

MAPK6-ChIP-F	CATCATCCAATCACCTCC	MAPK6-ChIP-R	GACTGATCAAGGCAGAAG
MAPKAPK2-ChIP-F	GAGAGCTTAACACCTCTG	MAPKAPK2-ChIP-R	GTGTTCAAGAAGCACACCTG
MAPK4-ChIP-F	CAATCCCCTCAACTACCT	MAPK4-ChIP-R	GGAGTTTGGTCTCCTAAC
MAP3K2-ChIP-F	CTCAGGAGTTTGAGACCAGT	MAP3K2-ChIP-R	AGGACTCAAGCGATCTTC
BRAF-ChIP-F	AGACAGAGTCTTGCTCTGTC	BRAF-ChIP-R	GTACTCCCAGCTATTGAGGA
RAF1-ChIP-F	CATCTGAGCATCTCACTAGC	RAF1-ChIP-R	CCTTGTCAGGGAGTTTAG
KRAS-ChIP-F	CTGACCCATAATCTTGCACC	KRAS-ChIP-R	GATACCTGCTAGGAAGCA
SOS2-ChIP-F	GTGAGGAAAGGTCTTCTG	SOS2-ChIP-R	GTGTCACTATTATCTGCTAGC

RT-QPCR primers

MAPKAPK2-QPCR-F	CTGCAGATCAAGAAGAACGC	MAPKAPK2-QPCR-R	GTCCTGAAGCATTTTGAGGG
KRAS-QPCR-F	GATGGAGAAACCTGTCTC	KRAS-QPCR-R	CAGCTCCAACTACCACAAG
MAPK6-QPCR-F	GCAAATGTACTGCACAGAG	MAPK6-QPCR-R	CAGAAAGATGACCCTTATGGG
SOS2-QPCR-F	GAAAGACAGTATCTACGGG	SOS2-QPCR-R	CTAAGGGATGAGGACTGCTTTC
MAPK4-QPCR-F	GTGATGCCTTCCTTTGTC	MAPK4-QPCR-R	TCCATGGGGTTAAAGGTCAG
MAPK3K2-QPCR-F	CTCAGGAAGTTGTCCATCAC	MAPK3K2-QPCR-R	CCTTCTTGGATATGTTCCTCC
BRAF-QPCR-F	ATAGAGGCCCTATTGGAC	BRAF-QPCR-R	GTTGGAGTGCATCTAGCTTG
RAF1-QPCR-F	CAGTGGTCAATGTGCGAAATG	RAF1-QPCR-R	CCTTTGTGTTCGTGGAGAAG

ChIP-QPCR primers

MAPK6-ChIP-F	CATCATCCAATCACCTCC	MAPK6-ChIP-R	GACTGATCAAGGCAGAAG
MAPKAPK2-ChIP-F	GAGAGCTTAACACCTCTG	MAPKAPK2-ChIP-R	GTGTTCAAGAAGCACACCTG
MAPK4-ChIP-F	CAATCCCCTCAACTACCT	MAPK4-ChIP-R	GGAGTTTGGTCTCCTAAC
MAP3K2-ChIP-F	CTCAGGAGTTTGAGACCAGT	MAP3K2-ChIP-R	AGGACTCAAGCGATCTTC
BRAF-ChIP-F	AGACAGAGTCTTGCTCTGTC	BRAF-ChIP-R	GTACTCCCAGCTATTGAGGA
RAF1-ChIP-F	CATCTGAGCATCTCACTAGC	RAF1-ChIP-R	CCTTGTCAGGGAGTTTAG
KRAS-ChIP-F	CTGACCCATAATCTTGCACC	KRAS-ChIP-R	GATACCTGCTAGGAAGCA
SOS2-ChIP-F	GTGAGGAAAGGTCTTCTG	SOS2-ChIP-R	GTGTCACTATTATCTGCTAGC

RT-QPCR primers

MAPKAPK2-QPCR-F	CTGCAGATCAAGAAGAACGC	MAPKAPK2-QPCR-R	GTCCTGAAGCATTTTGAGGG
KRAS-QPCR-F	GATGGAGAAACCTGTCTC	KRAS-QPCR-R	CAGCTCCAACTACCACAAG
MAPK6-QPCR-F	GCAAATGTACTGCACAGAG	MAPK6-QPCR-R	CAGAAAGATGACCCTTATGGG
SOS2-QPCR-F	GAAAGACAGTATCTACGGG	SOS2-QPCR-R	CTAAGGGATGAGGACTGCTTTC
MAPK4-QPCR-F	GTGATGCCTTCCTTTGTC	MAPK4-QPCR-R	TCCATGGGGTTAAAGGTCAG
MAPK3K2-QPCR-F	CTCAGGAAGTTGTCCATCAC	MAPK3K2-QPCR-R	CCTTCTTGGATATGTTCCTCC
BRAF-QPCR-F	ATAGAGGCCCTATTGGAC	BRAF-QPCR-R	GTTGGAGTGCATCTAGCTTG
RAF1-QPCR-F	CAGTGGTCAATGTGCGAAATG	RAF1-QPCR-R	CCTTTGTGTTCGTGGAGAAG
